# FBXW7 tumor suppressor regulation by dualspecificity tyrosine-regulated kinase 2

**DOI:** 10.1038/s41419-023-05724-0

**Published:** 2023-03-18

**Authors:** Rafael Jiménez-Izquierdo, Rosario Morrugares, Lucía Suanes-Cobos, Alejandro Correa-Sáez, Martín Garrido-Rodríguez, Laura Cerero-Tejero, Omar M. Khan, Susana de la Luna, Rocío Sancho, Marco A. Calzado

**Affiliations:** 1 Instituto Maimónides de Investigación Biomédica de Córdoba (IMIBIC), Córdoba, Spain; 2 Departamento de Biología Celular, Fisiología e Inmunología, Universidad de Córdoba, Córdoba, Spain; 3 Hospital Universitario Reina Sofía, Córdoba, Spain; 4 Hamad Bin Khalifa University, College of Health and Life Sciences Qatar Foundation, Education City, Doha, Qatar; 5 Centre for Genomic Regulation (CRG), The Barcelona Institute of Science and Technology (BIST), 08003 Barcelona, Spain; 6 Centro de Investigación Biomédica en Red en Enfermedades Raras (CIBERER), Barcelona, Spain; 7 Universitat Pompeu Fabra (UPF), 08003 Barcelona, Spain; 8 Institució Catalana de Recerca i Estudis Avançats (ICREA), 08010 Barcelona, Spain; 9 Centre for Stem Cells and Regenerative Medicine, King’s College London, London, SE10 9RT UK; 10 Department of Medicine III, University Hospital Carl Gustav Carus, Dresden, Germany

**Keywords:** Kinases, Cell signalling, Post-translational modifications, Mechanisms of disease, Tumour-suppressor proteins

## Abstract

FBXW7 is a member of the F-box protein family, which functions as the substrate recognition component of the SCF E3 ubiquitin ligase. FBXW7 is a main tumor suppressor due to its ability to control proteasome-mediated degradation of several oncoproteins such as c-Jun, c-Myc, Cyclin E1, mTOR, and Notch1-IC. FBXW7 inactivation in human cancers results from a somatic mutation or downregulation of its protein levels. This work describes a novel regulatory mechanism for FBXW7 dependent on the serine/threonine protein kinase DYRK2. We show that DYRK2 interacts with and phosphorylates FBXW7 resulting in its proteasome-mediated degradation. DYRK2-dependent FBXW7 destabilization is independent of its ubiquitin ligase activity. The functional analysis demonstrates the existence of DYRK2-dependent regulatory mechanisms for key FBXW7 substrates. Finally, we provide evidence indicating that DYRK2-dependent regulation of FBXW7 protein accumulation contributes to cytotoxic effects in response to chemotherapy agents such as Doxorubicin or Paclitaxel in colorectal cancer cell lines and to BET inhibitors in T-cell acute lymphoblastic leukemia cell lines. Altogether, this work reveals a new regulatory axis, DYRK2/FBXW7, which provides an understanding of the role of these two proteins in tumor progression and DNA damage responses.

## Introduction

FBXW7 (F-box and WD repeat domain-containing 7) is a subunit of the multicomponent RING-type E3 ligase complex SKP1-Cullin (CUL1)-F-box (SCF), which is responsible for substrate recognition within the complex [1]. FBXW7 is ubiquitously expressed and is involved in relevant cell processes such as DNA repair, cell cycle regulation, cell differentiation, and signal transduction [2, 3]. In mammals, three different isoforms with distinct subcellular localizations have been described: FBXW7α (nucleoplasm), FBXW7β (cytoplasmic), and FBXW7γ (nucleolar) [4–6]. The isoforms differ in their N-terminal regions, and the common core region contains the functional domains, including the dimerization domain, the F-box domain for recruitment to the SCF complex, and C-terminal WD40 domains involved in substrate recognition. Importantly, FBXW7 targets its substrates in a phosphorylation-dependent manner through interaction with a specific conserved phosphodegron sequence ((L)-X-pT/pS-P-(P)-X-pS/pT) [7–9]. FBXW7 is a critical tumor suppressor because it promotes the degradation of known oncoproteins including Cyclin E1 [10], c-Myc [11], MCL1 [12], mTOR [13], c-Jun [14], Notch1 [15], p53 [16] or SNAIL [17]. Loss or inactivation of FBXW7 results in the accumulation of these proteins, which increases tumorigenesis and chemotherapy resistance [2, 18, 19]. Deletions of the *FBXW7* chromosomal region (4q31) are detected in >30% of human cancers and the gene is mutated in 6%, being *FBXW7* the most mutated ubiquitin-proteasome-related gene [20]. Furthermore, 43% of mutations occur in critical residues for substrate recognition (R465, R505, and R479) [21]. Regulatory mechanisms impacting FBXW7 protein accumulation have been described at different levels through transcription factors, epigenetic regulators, miRNAs, and lncRNAs, as well as interaction with regulatory proteins and post-transcriptional modifications, including ubiquitin-mediated autocatalytic degradation [22]. Thus, regulation of FBXW7 stability is crucially involved in tumor development.

DYRK2 (Dualspecificity tyrosine-phosphorylation-regulated kinase 2) is a Ser/Thr kinase that belongs to the CGMC kinases group [23]. Similarly to FBXW7, DYRK2 plays an important role in the regulation of processes such as cell growth, survival, and differentiation, with particular involvement in cellular responses to DNA damage and stress signals [24]. Thus, DYRK2 is a key regulator of DNA damage response pathways and stress signals, and it has been implicated in several human cancers with both oncogenic and tumor suppressor activities [24, 25]. In some cases, DYRK2-dependent phosphorylation results in alterations in the stability of its substrates as shown for c-Jun and c-Myc [26], Notch1 [27], HSF1 [28], CDC25A [29], GLI2 [30], SNAIL [31], Sirtuin 1 [32], mTOR1 [33], and TBK1 [34]. Though several DYRK2 substrates are well-known FBXW7 substrates, the existence of a crosstalk between DYRK2 and FBXW7 remains unexplored.

Here we report the identification of DYRK2 kinase as a novel negative regulator of FBXW7 protein stability. DYRK2 binds and phosphorylates FBXW7 leading to its ubiquitination-mediated degradation. We provide evidence that DYRK2 is involved in FBXW7 stability control in response to DNA damage, with important consequences on cell survival, and rendering cancer cells sensitive to the chemotherapy drug Paclitaxel and anti-cancer BET (Bromodomain and Extra-Terminal motif) inhibitors.

## Materials and methods

### Cell culture, transfection, and reagents

HEK-293T (wt/DYRK2−/−), MDA-MB-468 (wt/DYRK2−/−), MDA-MB-231 (wt/DYRK2−/−), HeLa (wt/DYRK2−/−), CHO, A549, and SW837 cells were maintained in Dulbecco’s Modified Eagle’s medium (DMEM). HCT116 (wt/FBXW7−/−), HT-29 cells were maintained in McCoy’s 5A medium while Jurkat and MOLT-4 cells were maintained in Roswell Park Memorial Institute (RPMI) medium. All cell lines media were supplemented with 10% fetal bovine serum (FBS) and 1% (v/v) penicillin/streptomycin (Sigma-Aldrich, St Louis, Missouri, USA). Cells were maintained in a humidified atmosphere at 37 ºC containing 5% CO_2_.

All cell lines were regularly tested for mycoplasma and cross-contamination. Cell lines’ authentication was performed by a multiplex PCR with Geneprint10 System (Promega, Madison, Wisconsin, USA). The generation of CRISPR/Cas9-cell lines was previously described [27, 28]. Transient transfections were carried out with Roti-Fect (Carl Roth, Karlsruhe, Germany). siRNAs to FBXW7, DYRK2, or control (Dharmacon, Lafayette, CO, USA) were transfected using Lipofectamine 2000 (Invitrogen, Waltham, Massachusetts, USA) according to the manufacturer’s instructions. Point mutants were produced by directed mutagenesis using the QuikChange II Site-Directed Mutagenesis Kit (Agilent Technologies, California, USA). The different reagents, mutagenesis primers, and plasmids employed are listed in Supplemental Tables 1–3, respectively.

### Western blotting (WB)

Cells were lysed in NP-40 buffer, proteins were resolved on SDS-PAGE gels, blotted to polyvinylidene difluoride/nitrocellulose membranes, and incubated with appropriated primary antibodies overnight at 4 ºC after being blocked with non-fat milk or bovine serum albumin in TBS-Tween. Membranes were washed with TBS-Tween and incubated with suitable secondary antibodies (fluorochome- or horseradish peroxidase (HRP)-tagged). For HRP, the detection was carried out using Clarity™ Western ECL Substrate (Bio-rad, California, USA). For immunofluorescence-based detection, the immuno-reactive bands were visualized with a ChemiDoc MP Imaging System (Bio-rad). Antibodies and buffer composition are listed in Supplemental Tables 1 and 4, respectively.

### Immunoprecipitation

Cells were washed in phosphate-buffered saline (PBS) and lysed in IP buffer. Cell lysates were pre-cleared with protein A/G Sepharose (Santa Cruz, California, USA) and immunoprecipitation was performed on a rotating wheel upon the addition of 1.5 μg of the indicated antibodies and 50 μl of protein A/G Sepharose beads. Immunoprecipitated proteins on beads were then washed five times in IP buffer and eluted in 1.5× SDS sample buffer, followed by WB. Antibodies and buffer composition is described in Supplemental Tables 1 and 4.

### Clonogenic survival assay

Transfected HEK-293T cells were seeded in 24-well plates at 70% confluence were treated with 2 μM Doxorubicin and then incubated for 24 h. Afterwards, 10^3^ treated cells were seeded in 6-well plates and incubated for 7 days. Cells were stained with Crystal violet and the number of colonies (accumulations of more than 50 cells) was analyzed using Image J software (http://imagej.nih.gov/ij/).

### Data analysis

Data are expressed as mean ± SD. Differences were analyzed by unpaired Student’s *t* test, and *P* < 0.05 was considered significant. Statistical analyses were performed using GraphPad Prism version 8.00 (GraphPad, San Diego, CA, USA). Images were analyzed and quantified using the ImageJ v1.45 software. Protein abundance in tumor tissue was obtained from The Human Protein Atlas database (https://www.proteinatlas.org/) [35] as antibody staining level (not detected, low, medium, and high) per patient. Data were accessed via the FireBrowse R package (https://github.com/mariodeng/FirebrowseR) [36]. Gene alteration frequencies were calculated using the Cancer Genome Atlas (TCGA) PanCancer dataset that includes 10 967 samples across 33 different tumor types [37]. To calculate the alteration frequencies, the number of samples containing a missense/nonsense mutation or a deep deletion for a given gene was divided by the total number of samples in each cancer type. Details for the generation of the 3D FBXW7 structure model and other methods employed in the article are provided in Supplemental Methods.

## Results

### DYRK2 modulates FBXW7 protein levels

**Fig. 1:**
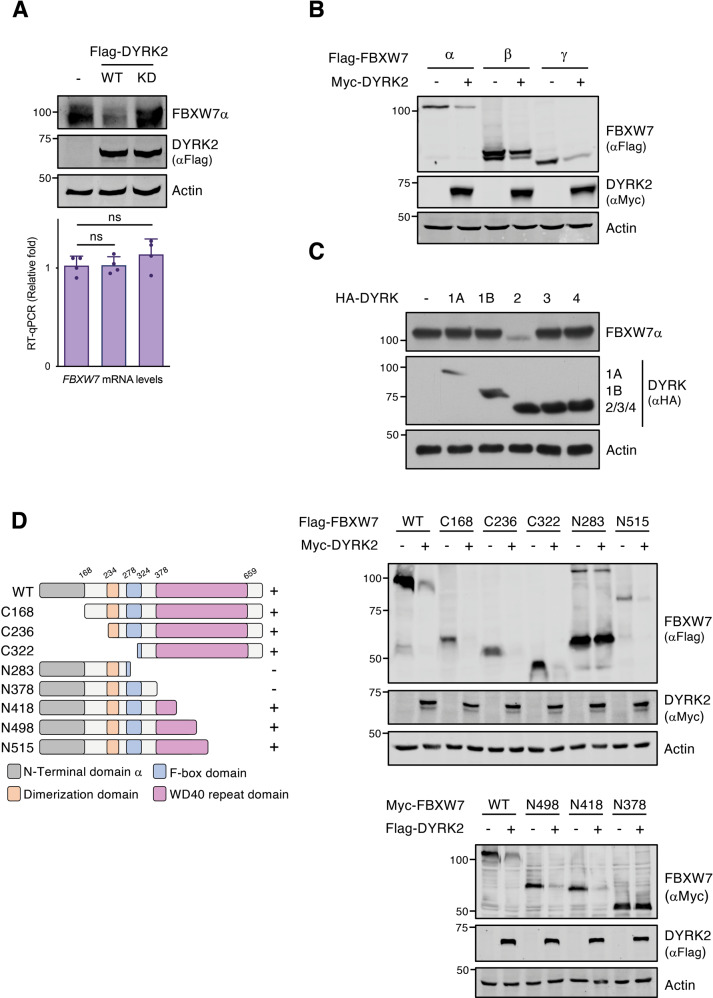
FBXW7 protein levels are regulated by DYRK2. **A** HEK-293T cells were transfected (2 × 10^5^ cells/35-mm dish) with 0.4 μg of either the control expression vector, wild type (WT) or kinase-dead (KD, mutation in the ATP binding site K178M) DYRK2 expression vectors and cells harvested after 48 h. One fraction was used to analyze endogenous FBXW7α and exogenously expressed DYRK2 protein levels by immunoblotting (upper panel, a representative experiment is shown, *n* = 4), while another aliquot was used to analyze FBXW7α mRNA levels (lower panel, the graph shows relative RNA levels determined by RT-qPCR with mock-transfected cells set up as 1; mean ± SD, *n* = 4; ns, not significant). **B** HEK-293T cells (2 × 10^5^ cells/35-mm dish) were transfected with plasmids to express FBXW7 isoforms α, β, and γ (0.2 μg) together with Myc-DYRK2 (0.2 μg) or the control expression vector (0.2 μg). Cells were lysed after 48 h and protein expression analyzed by WB. **C** Endogenous FBXW7α protein expression was evaluated by WB in HEK-293T cells expressing the five different human DYRK family members. **D** The indicated Flag-tagged truncated versions of FBXW7α were transiently overexpressed in the presence or absence of Myc-DYRK2 in HEK-293T cells. The schematic representation of FBXW7α indicates the positions of the N-terminal domain (grey), the dimerization domain (orange), the F-box domain (blue), and the region containing the WD40 domains (pink). Note: a representative experiment is shown in each panel of 3–4 performed.

Some of the most relevant DYRK2 substrates described so far, such as c-Jun, c-Myc, or Notch1-IC are targets of FBXW7. To analyze the existence of possible crosstalk between DYRK2 and FBXW7, we first assessed the effect of DYRK2 exogenous expression on FBXW7. FBXW7α protein levels, but not mRNA levels, were reduced in the presence of DYRK2 in a kinase activity-dependent manner (Fig. 1A and SFig. 1A), suggesting a post-transcriptional effect. The effect of DYRK2 on FBXW7 was isoform independent as DYRK2 overexpression reduced protein levels of FBXW7α, FBXW7β, and FBXW7γ (Fig. 1B). Other F-box family members such as FBXW1 (β-TrCP) and SKP2 were analyzed. DYRK2 expression reduced FBXW1 protein levels (less than FBXW7), not affecting the levels of SKP2 (SFig. 1B). On the other hand, the DYRK2 effect was specific, since none of the other members of the human DYRK subfamily (DYRK1A, DYRK1B, DYRK3, and DYRK4) showed an effect on FBXW7α accumulation (Fig. 1C and SFig. 1C). Moreover, a region in FBXW7 (aa 378-418) appeared to be necessary for the DYRK2-mediated effect, as indicated by the response of different FBXW7α truncated versions to DYRK2-induced protein reduction (Fig. 1D).

**Fig. 2:**
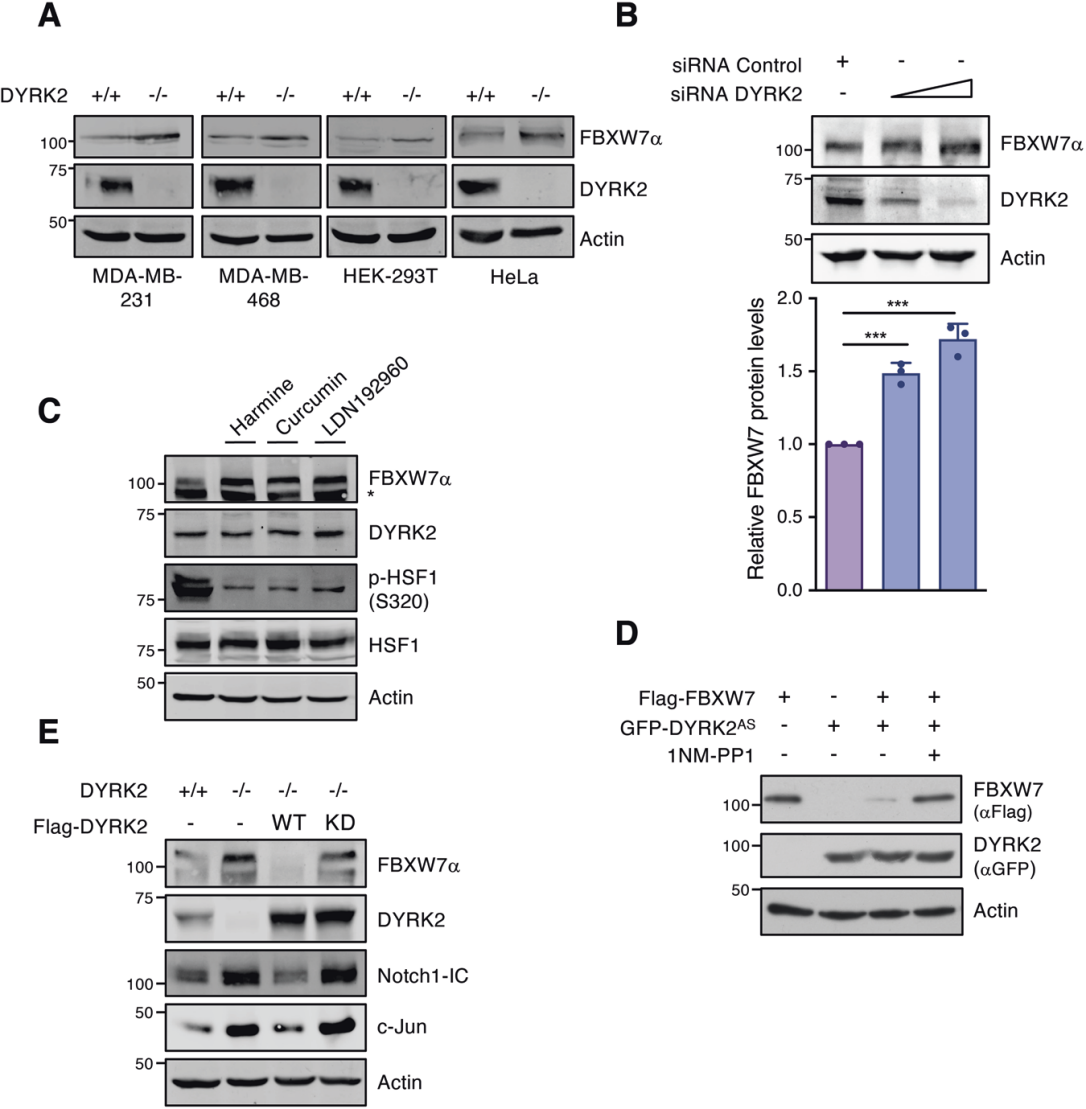
Stable or acute DYRK2 depletion modulate FBXW7 protein stability. **A** FBXW7α and DYRK2 protein levels were analyzed in the indicated cell lines WT (+/+) and knockout (−/−) for DYRK2. **B** *DYRK2* was silenced in HEK-293T cells using increasing concentration of specific siRNAs and FBXW7α endogenous levels were analyzed. The bar graph shows the quantification of FBXW7α (normalized by Actin) with that of the siRNA Control set as 1 (mean ± SD, *n* = 3; ***P < 0.001). **C** A549 cells were treated with 10 μM Harmine for 12 h, 5 μM LDN192960 for 2 h, or 5 μM Curcumin for 6 h, and the indicated proteins analyzed by WB. Phosphorylation of HSF1 has been used as a marker for the inhibition. *, non-specific. **D** HEK-293T cells were transfected using the indicated plasmids and treated with the PP1 analog 1NM-PP1 (3 mM) for 3 h at 48 h post-transfection. Protein levels were analyzed by WB. **E** HEK-293T cells WT (+/+) and knockout (−/−) for DYRK2 were transfected with Flag-DYRK2 either wild type (WT) or kinase-dead (KD) plasmids. Protein expression of endogenous FBXW7α, DYRK2, Notch1-IC, and c-Jun were evaluated by WB. Note: a representative experiment is shown in each panel of 3–4 performed.

To validate these results, the effect of stable or acute DYRK2 depletion was analyzed using *DYRK2* knockout cell lines generated by CRISPR/Cas9 (Fig. 2A) or transient reduction of DYRK2 by siRNA-dependent silencing (Fig. 2B). *DYRK2* depletion by any of these methods resulted in increased FBXW7α protein levels. The dependence on the DYRK2 kinase activity was further evaluated with DYRK2 small molecule inhibitors. Treatment with a general DYRK inhibitor like Harmine [38], the potent selective DYRK2 inhibitor LDN192960 [39], or Curcumin [40] significantly increased FBXW7α protein levels (Fig. 2C). HSF1 phosphorylation at S320 [28] was included to verify inhibition of endogenous DYRK2 activity. Likewise, whereas an analogue sensitive DYRK2 mutant (DYRK2-AS) responsive to pyrazolo[3,4-*d*]pyrimidine-based (PP1) inhibitors by mutation of a gatekeeper residue in the kinase domain [28] induced a reduction in FBXW7 levels, the effect was not observed in the presence of the AS-kinase inhibitor 1NM-PP1 (Fig. 2D). Similar results were obtained by reintroducing DYRK2 WT in *DYRK2* knockout cells, with relevant consequences on substrates of both proteins, such as c-Jun and Notch1-IC. In addition, this effect was not observed with a kinase-dead mutant version (Fig. 2E).

**Fig. 3:**
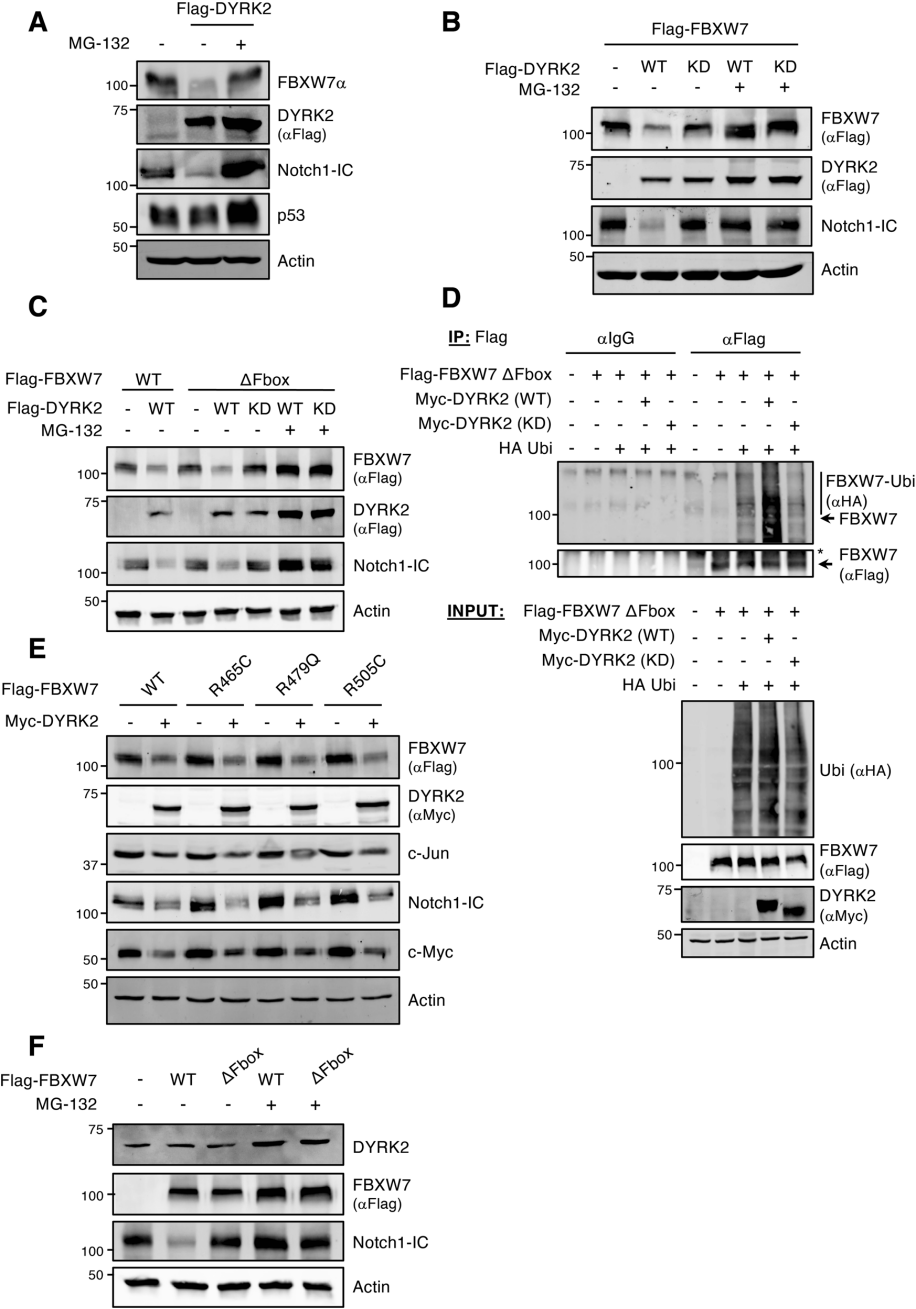
FBXW7 degradation by DYRK2 is proteasome and DYRK2 kinase activity-dependent and independent of the FBXW7 ubiquitin ligase activity. **A** A549 cells were treated with the proteasome inhibitor MG-132 (10 μM) for 12 h prior to analysis. Cell extracts were analyzed by WB with the indicated antibodies. **B** HEK-293T cells were transfected to express the indicated protein combination and treated with 10 μM MG-132 for 12 h. Protein expression was evaluated by WB. **C** Extracts from transfected HEK-293T cells with the indicated plasmids and treated with MG-132 (10 μM) during 12 h were analyzed by WB. **D** Transfected HEK-293T cells with the indicated plasmids were treated with MG-132 (10 μM) for 12 h. Flag-FBXW7α-ΔFbox was purified by anti-Flag immunoprecipitation and ubiquitinated FBXW7 was detected by WB. *, non-specific (**E**) HEK-293T cells transfected with Flag-FBXW7α WT or the indicated Flag-FBXW7 mutant versions, alone or together with Myc-DYRK2 WT were analyzed by WB with the indicated antibodies. **F** HEK-293T cells were transfected with either Flag-FBXW7 or Flag-FBXW7-ΔFbox expression plasmids and then treated with MG-132 (10 μM) for 12 h. Cell extracts were analyzed by WB with the indicated antibodies. Note: a representative experiment is shown in each panel of 3–4 performed.

The post-transcriptional nature of the DYRK2 effect on FBXW7 suggested alterations in protein stability. Indeed, DYRK2 expression reduced FBXW7 half-life (SFig. 1D, E). In agreement, the proteasome inhibitor MG-132 blocked DYRK2-induced FBXW7 reduction (Fig. 3A, B), indicating a mechanism mediated by the ubiquitin-proteasome system. Internal controls such as Notch1-IC and p53 were included to verify that MG-132 is properly working (Fig. 3A). FBXW7 turnover can be controlled by autocatalytic-induced degradation [22]. Thus, we used an FBXW7α ubiquitin ligase deficient version (FBXW7-ΔFbox), without critical residues of F-box domain (Δ284-288 aa), to verify whether DYRK2 promotes this event. Interestingly, the mutant protein responded to DYRK2 in the same way as the WT protein (Fig. 3C). Similar results were obtained with a full F-box deletion mutant to eliminate possible residual functionality (SFig. 1F) or in the presence of a dominant-negative form of SCF complex scaffold Cullin-1 (SFig. 1G), disabling the subsequent complex formation. These results support that DYRK2-dependent degradation has been produced by a mechanism independent of FBXW7α ubiquitin ligase activity. Next, we investigated whether DYRK2 kinase activity regulates FBXW7α ubiquitination. We observed that wild-type DYRK2, but not a kinase-dead mutant, greatly increased the poly-ubiquitination of FBXW7-ΔFbox (Fig. 3D and SFig. 1H). We also explored the behavior of several cancer hotspot FBXW7 variants with inactivating mutation, R465C, R479Q, and R505C [41]. No differences were observed in the response to DYRK2 as compared with FBXW7 WT, with similar consequences on substrates of such as c-Jun, Notch1-IC, and c-Myc (Fig. 3E). Finally, we also determined whether FBXW7α could be targeting DYRK2 for degradation as part of the crosstalk between the two proteins. However, no significant alterations in DYRK2 accumulation were observed upon overexpression of FBXW7α (Fig. 3F). Altogether, these results demonstrate that DYRK2 negatively regulates FBXW7 levels by the ubiquitin-proteasome pathway. This mechanism is DYRK2 kinase activity-dependent and independent of the FBXW7α ubiquitin ligase activity.

### DYRK2 phosphorylates FBXW7α

**Fig. 4:**
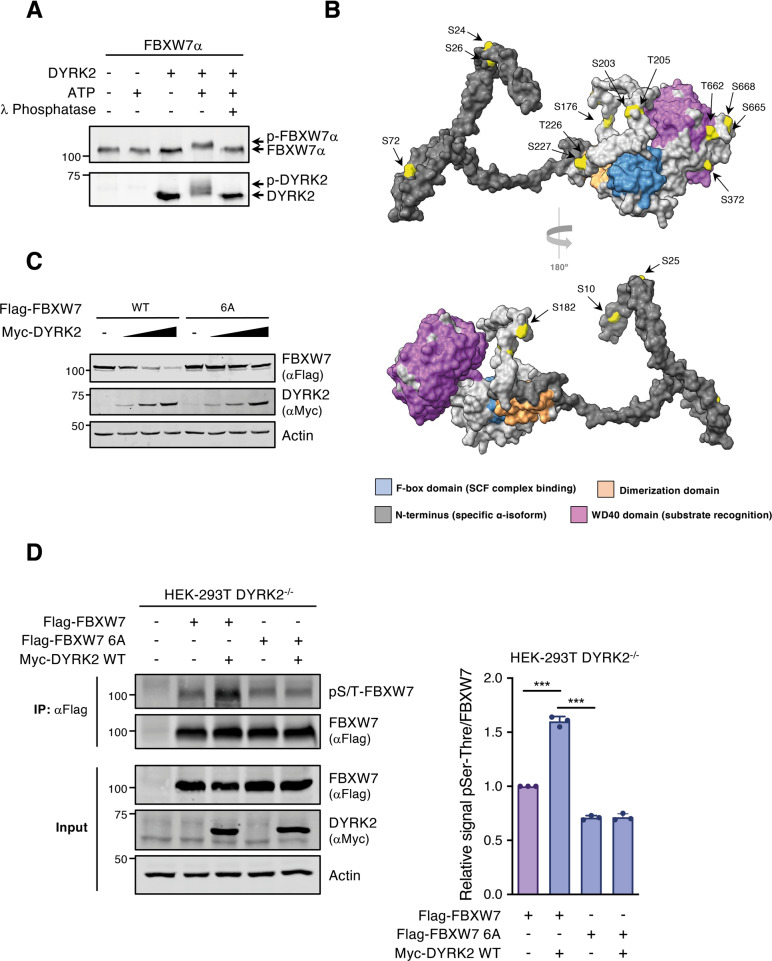
DYRK2 phosphorylates FBXW7. **A** FBXW7α and DYRK2 recombinant proteins were incubated in the presence of ATP and λ-phosphatase and analyzed by WB. Note the electrophoretic mobility shifts in the two proteins, which in the case of DYRK2 are due to autophosphorylation [29]. **B** Surface-filling models of FBXW7α 3D structure prediction shown from different angles; the color code is for the different domains and the phosphorylated amino acids identified by MS are in yellow. **C** Extracts from cells expressing Flag-FBXW7α WT or a mutant version with six S/T-to-A changes (6 A: S176, S182, T205, S227, S372, and S688), either alone or together with increasing amounts of Myc-DYRK2, were analyzed by WB. **D** HEK-293T DYRK2-KO cells were transfected with Flag-FBXW7α WT or Flag-FBXW7 6 A mutant in the presence or absence of Myc-DYRK2 as indicated, followed by anti-Flag immunoprecipitation. Cells were previously treated with MG-132 (10 μM) to avoid FBXW7 degradation. Total FBXW7α was detected with a Flag antibody and phosphorylated FBXW7α with an antibody detecting phospho-serine and phospho-threonine residues (pS/T). The bar graph shows the ratio phospho-FBXW7/total FBXW7 in the immunoprecipitates, set as 1 in the absence of Myc-DYRK2 (mean ± SD, *n* = 3; **P < 0.01). Note: a representative experiment is shown in each panel of 3–4 performed.

The requirement of DYRK2 kinase activity suggests the possibility of FBXW7α being a DYRK2 substrate. To test this hypothesis, we performed in vitro kinase assays (IVK) with purified proteins commercially available. As shown in Fig. 4A, incubation with DYRK2 in the presence of ATP caused the appearance of FBXW7α bands with lower electrophoretic mobility, which disappeared in the presence of λ−phosphatase, thus indicating direct FBXW7α phosphorylation by DYRK2. In agreement, loss of the DYRK2-induced FBXW7α lower mobility bands was also observed when the IVK was performed in the presence of DYRK2 inhibitor LDN192960 (SFig. 2A).

To identify the sites in FBXW7α that are phosphorylated by DYRK2, we used mass spectrometry analysis on purified FBXW7α phosphorylated by DYRK2 in vitro. With a coverage of around 70% of the protein (SFig. 2B), the analysis identified 19 phosphorylated residues in FBXW7α (Fig. 4B and SFig. 2B, C). These residues are distributed throughout the FBXW7α sequence (SFig. 2D), and several of them have been already identified in low- and high-throughput in vivo experiments as listed in Phosphosite (https://www.phosphosite.org). All the sites are conserved in FBXW7 orthologs from human to *Xenopus laevis* (SFig. 2D). Almost all sites, except S396, S688, and S516, are exposed when placed on the FBXW7α 3D structure (Fig. 4B and SFig. 2E). In addition, whereas several of them could be DYRK2 target sites common to all FBXW7 isoforms, some others are located within the specific region of the FBXW7α isoform (Fig. 4B and SFig. 2D).

To identify the residues involved in the FBXW7 regulation by DYRK2, we first generated FBXW7α versions with each serine and threonine present in the region aa 378-418 mutated to alanine (T385, S398, T402, T410, and T416). As shown in SFig. 2F, all single mutants were negatively regulated by DYRK2 expression to a similar extent as the WT protein. Next, we evaluated the importance of the residues identified by mass spectrometry analysis. We also included two residues not detected in the analysis, S18 and S349 (lack of coverage), but previously described as relevant for FBXW7α regulation [42, 43]. As shown in SFig. 2G, all single mutants and the triple mutant T662/S665/S668A were negatively regulated by DYRK2 expression to a similar extent as the WT protein. On the contrary, an FBXW7α variant with the six most relevant residues based on their similarity to the phosphorylation consensus sequence of DYRK2 mutated to alanine (FBXW7-6A: S176, S182, T205, S227, S372, S688) were highly resistant to DYRK2-induced degradation (Fig. 4C) without affecting its ability to interact with DYRK2 (SFig. 2H). Analysis of FBXW7α phosphorylation levels using a phospho-serine/threonine antibody revealed a significant increase in signal when DYRK2 WT was reintroduced into *DYRK2* knockout cells (Fig. 4D). This increase was not observed with the FBXW7-6A mutant, further supporting that DYRK2 phosphorylates FBXW7 in cells. Together, these results indicate that DYRK2 directly phosphorylates FBXW7 at several residues, and that phosphorylation at more than one of these sites is required for DYRK2 to induce FBXW7 degradation.

### DYRK2 interacts and co-localizes with FBXW7

**Fig. 5:**
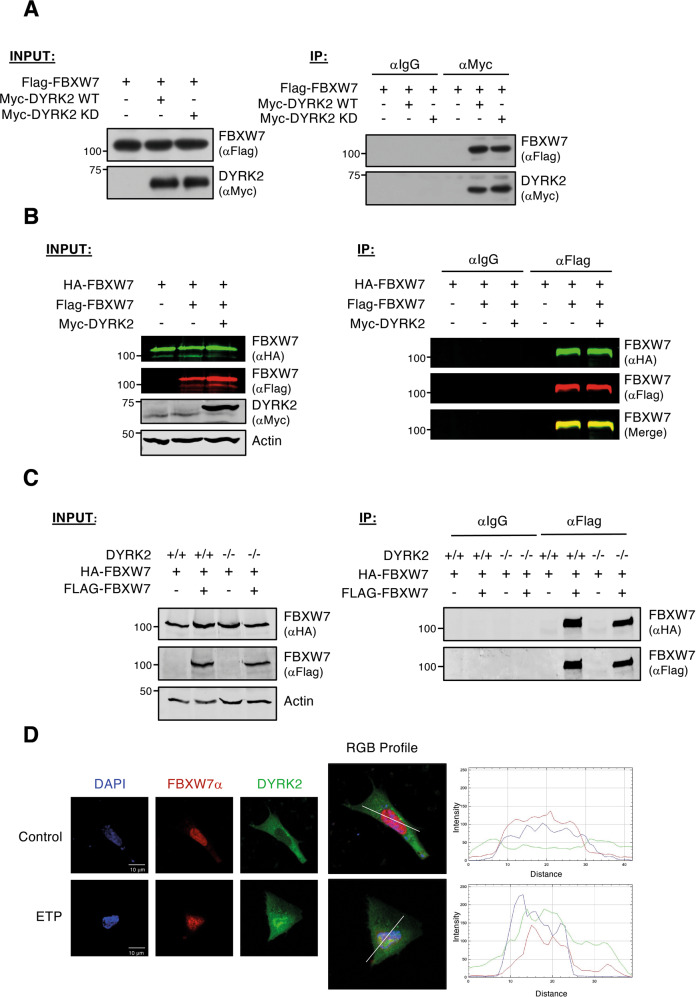
DYRK2 interacts and co-localizes with FBXW7. **A** HEK-293T cells were co-transfected with Flag-FBXW7 and Myc-DYRK2 WT or kinase-dead (KD) expression plasmids. Cells were treated with MG-132 (10 μM) to stabilize FBXW7. Cell extracts were subjected to immunoprecipitation with a Myc antibody or IgGs control. Both the input (5%) and the immunoprecipitates (IP) were analyzed by WB. **B** HEK-293T cells expressing HA-FBXW7 and Flag-FBXW7 with or without Myc-DYRK2 were used in immunoprecipitation experiments with a Flag antibody. Cells were treated with MG-132 (10 μM) to avoid FBXW7 degradation. Both the lysate (5%) (INPUT) and the immunoprecipitate (IP) were analyzed by WB. **C** HEK-293T WT (+/+) and DYRK2 (−/−) cells were transfected with HA-FBXW7 and Flag-FBXW7 plasmids and treated with MG-132 (10 μM) for 12 h prior to extract preparation. Both the lysate (5%) (INPUT) and the immunoprecipitates (IP) were analyzed by WB. **D** CHO cells were transfected with a GFP-DYRK2 plasmid and analyzed for the subcellular localization of DYRK2 (GFP-fluorescence) and endogenous FBXW7α (indirect immunofluorescence) by confocal microscopy in cells treated with ETP (10 μM) or vehicle for 6 h and with MG-132 (10 μM) for the last 4 h to promote FBXW7 stabilization. DNA was stained with DAPI. Overlapping localization is shown in yellow. Fluorescence intensity profiles through the white line indicate GFP-DYRK2 and FBXW7 cellular localization in both control and DNA damage conditions. Pearson’s coefficient (0.377) and thresholded Manders’ coefficients A and B (*A* = 0.4515; *B* = 0.4007) were calculated for DNA damage situation. Note: a representative experiment is shown in each panel of 3–4 performed.

At this stage, our results indicate that FBWX7 can be a possible DYRK2 substrate. So, we wondered whether this functional interaction was depending on the formation of a stable complex between the two proteins. The results of co-immunoprecipitation experiments showed efficient interaction between the two proteins (Fig. 5A). The interaction was independent of the DYRK2 catalytic activity (Fig. 5A), ruling out that the lack of effect of DYRK2 KD on FBXW7 stability was due to the inability of the kinase-inactive version to interact with the target.

Phosphorylation of FBXW7 has been shown to impact dimerization and subcellular localization [42, 44]. Therefore, we investigated whether DYRK2 could affect FBXW7 dimerization, and proved that it was not altered by the forced expression of DYRK2 (Fig. 5B) or by its absence (Fig. 5C). Next, we analyzed whether DYRK2-induced changes in the subcellular localization of FBXW7, but no changes were observed in the nuclear localization of endogenous FBXW7α (Fig. 5D) or exogenously expressed FBXW7α (SFig. 3A) by DYRK2 overexpression. As previously shown, the nuclear localization of DYRK2 substantially increased after exposure to DNA damage-inducing chemotherapeutic agents such as etoposide (ETP), and a co-localization profile between DYRK2 and FBXW7α was clearly observed under these conditions (Fig. 5D and SFig. 3A). These results demonstrate the direct interaction and co-localization between DYRK2 and FBXW7, which is increased in response to chemotherapy agents.

### DYRK2 modulates FBXW7 biological activity

**Fig. 6:**
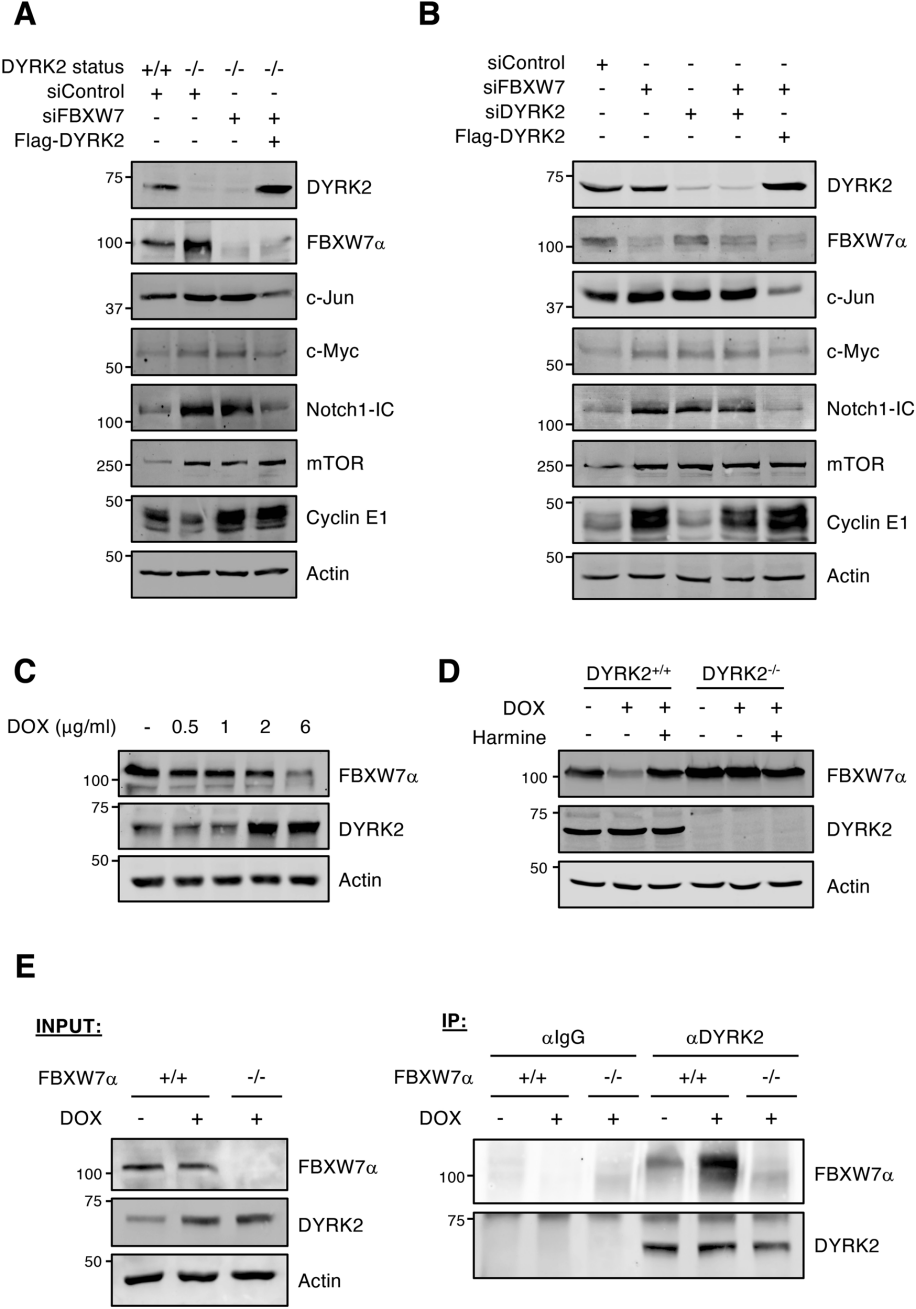
DYRK2 modulates FBXW7 activity. **A** HEK-293T WT (+/+) or DYRK2-KO (−/−) cells were transfected with the indicated siRNAs or plasmids, and the indicated endogenous proteins were analyzed by WB. **B** HEK-293T cells were transfected with the indicated siRNAs or plasmids, and the endogenous levels of the specified proteins were analyzed by WB. **C** HEK-293T cells were treated with increasing concentrations of DOX for 12 h, and endogenous FBXW7α and DYRK2 analyzed by WB. **D** HEK-293T cells and derived DYRK2-KO cells were treated with DOX (2 μg/ml) or vehicle for 12 h in the presence or absence of Harmine (5 μM), and endogenous DYRK2 and FBXW7α analyzed by WB. **E** HCT116 cells WT (+/+) and knockout (−/−) for FBXW7 were treated with DOX (2 μg/ml) or with vehicle for 12 h. MG-132 (10 μM) was included to stabilized FBXW7. Control and treated extracts (INPUT) were immunoprecipitated with a DYRK2 antibody or control IgGs and the presence of DYRK2 and FBXW7 in the immunoprecipitates analyzed by WB (IP). Note: a representative experiment is shown in each panel of 3–4 performed.

To determine the impact of DYRK2 on FBXW7 biological activity, we analyzed relevant FBXW7 substrates such as c-Jun, c-Myc, Notch1-IC, mTOR, and Cyclin E1. As expected, FBXW7 silencing increased abundance of all these proteins (Fig. 6B). In agreement with published results [26, 27, 33], similar results were obtained by DYRK2 depletion except for Cyclin E1 (Fig. 6A, B). Noteworthy, DYRK2 phosphorylates residues within the FBXW7 phosphodegron of c-Myc, c-Jun, mTOR, and Notch1-IC [26, 27, 33], with Cyclin E1 not being described as a DYRK2 substrate to date (SFig. 4A). Depletion of DYRK2 elevated c-Jun, c-Myc, Notch1-IC, and mTOR protein levels regardless of FBXW7 protein levels being higher than in control cells (Fig. 6A), suggesting that DYRK2 role is placed upstream of FBXW7. The results might be also interpreted as a requirement of FBXW7 phosphorylation by DYRK2 for its activation and/or substrate choice. Moreover, when both proteins were depleted, the target protein levels remained high. Interestingly, c-Jun, c-Myc, and Notch1-IC, but not mTOR or Cyclin E1, protein levels were reduced in the DYRK2 rescuing conditions despite low FBXW7 levels (Fig. 6A, B), indicating the existence of FBXW7-independent regulatory mechanisms for these three substrates. To reinforce this possibility, we analyzed the effect of DYRK2 on Notch1-IC and Cyclin E1 levels, comparing WT with FBXW7 knockout cells. As shown in Supplemental Fig. 4B, in cells lacking FBXW7 a decrease in Notch1-IC is still observed in response to DYRK2, being substantially smaller than in the wild-type version. In addition, the results for Cyclin E1 agreed with FBXW7-dependence and DYRK2-independence for the regulation of the stability of this protein (SFig. 4B).

Due to the increased co-localization of both proteins and their relevant role in cellular responses to DNA damage, we were poised to study the DYRK2/FBXW7 axis in cells exposed to inducers of double-strand breaks. Treatment with the chemotherapy agent Doxorubicin (DOX) caused a gradual increase in DYRK2 levels, which was accompanied by a decrease in FBXW7 protein accumulation (Fig. 6C). Notably, no effect on FBXW7 accumulation in response to DOX was observed in cells lacking DYRK2 (Fig. 6D); moreover, FBXW7 levels were recovered in the presence of the inhibitor Harmine (Fig. 6D), supporting a kinase activity-dependent effect. In fact, co-immunoprecipitation experiments proved that the interaction between the two proteins occurred in the presence of DOX (Fig. 6E and SFig. 4C). These results strongly support the involvement of DYRK2 in the regulation of FBXW7 accumulation in response to the genotoxic agent.

### DYRK2 regulates cell reproductive death after treatment with cytotoxic agents via FBXW7

**Fig. 7:**
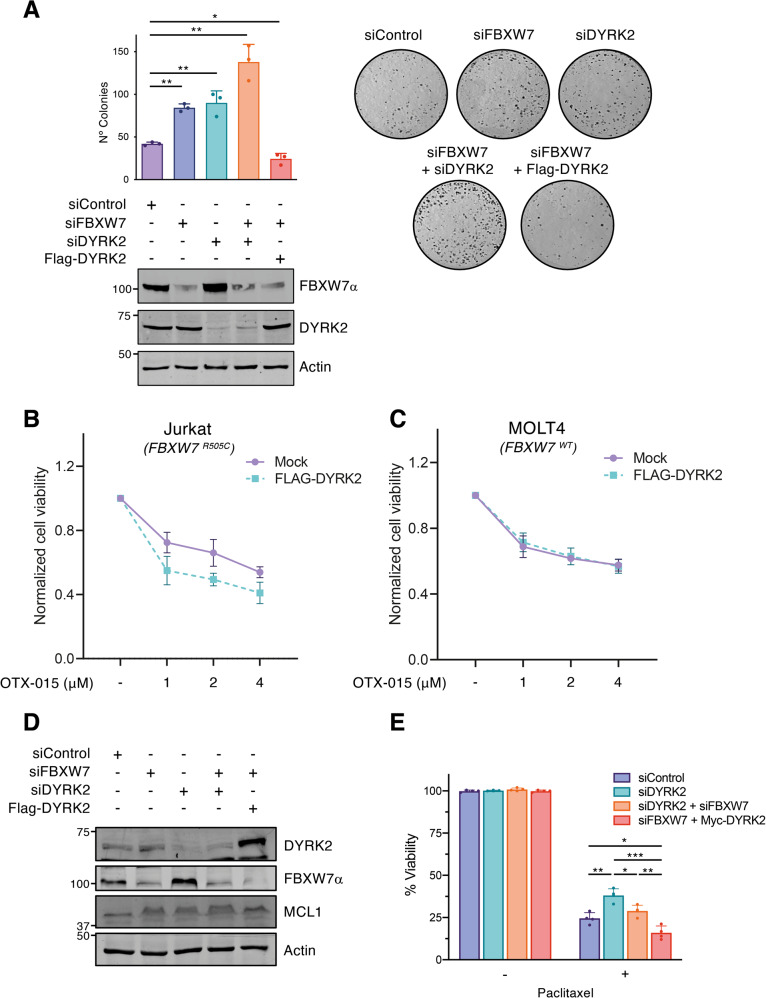
DYRK2 regulates chemotherapy resistance via FBXW7. **A** Survival clonogenic assay of HEK-293T cells in the conditions indicated and treated with DOX (2 μM) for 24 h prior to cell plating in diluted conditions. Quantification of the data is shown in the bar graph (mean ± SD, *n* = 3; *P < 0.05, **P < 0.01). Expression of the indicated proteins was assessed by WB in parallel samples. **B** Jurkat cells (*FBXW7* ^*R505C*^) or **C** MOLT-4 cells (*FBXW7* ^*WT*^) were transfected with Flag-DYRK2, and treated with OTX-015 for 48 h at the concentrations indicated. Viability was analyzed by XTT assay. **D** HCT116 cells were transfected with the indicated siRNAs or plasmids, and the endogenous levels of the specified proteins were analyzed by WB. **E** Cell viability of HCT116 cells transfected with the indicated siRNAs or plasmids was evaluated by MTT assay in the presence or absence of Paclitaxel (100 nM) treatment for 72 h and presented as relative to the untreated control (mean ± SD, *n* = 4; *P < 0.05, **P < 0.01, ***P < 0.001). Note: a representative experiment is shown in each panel out of 3–4 performed.

FBXW7 protein expression has been inversely correlated with poor prognosis and resistance to chemotherapy in a variety of tumor types [19, 45]. Therefore, the cytotoxic effect of the DYRK2 and FBXW7 functional interaction was evaluated in this context. As shown in Fig. 7A, a pro-cytotoxic effect of DYRK2 and FBXW7 in response to DOX was observed based on the increase in the number of colonies when either protein was depleted; the protective effect was enhanced when both proteins were depleted together. Furthermore, DYRK2 overexpression in conditions of reduced expression of FBXW7 significantly reduced the number of colonies with respect to the control. These results support the participation of the DYRK2/FBXW7 axis in the survival potential of cells in response to genotoxic agents, not excluding a further regulatory effect on cells' proliferative capacity.

In the same context, recent evidence has revealed that mutation or lack of expression of FBXW7 contributes to BET inhibitors’ resistance, as in the case of T-cell leukemia cells by disruption of c-Myc degradation and its subsequent accumulation [46]. As shown in Fig. 6A, DYRK2 overexpression triggers c-Myc degradation in an FBXW7-independent manner. Therefore, we wondered whether DYRK2 could resensitize FBXW7 mutant cell lines to BET inhibitors. In order to achieve our objective, the Jurkat cell line was utilized as a model system that possesses the FBXW7 R505C mutation. This specific mutation hinders the degradation of c-Myc by FBXW7 [46]. Indeed, ectopic DYRK2 expression showed a clear tendency to sensitize Jurkat cells to the BET inhibitor OTX-015 (Fig. 7B). Interestingly, this sensitization was not observed when using a T-cell leukemia cell line such as MOLT-4 carrying FBXW7 WT alleles (Fig. 7C) under similar experimental conditions (DYRK2 expression and transfection efficiency, SFig. 4D).

Equally important, mounting evidence has indicated the involvement of aberrant expression of FBXW7 in tumorigenesis. Indeed, cancer-associated FBXW7 mutations cause resistance to anti-tubulin chemotherapeutics via accumulation of MCL1 protein [47]. Therefore, we investigated the effect of DYRK2 depletion on MCL1 expression in the HCT116 cell line. As shown in Fig. 7D, and concurring with published results, FBXW7 silencing resulted in the upregulation of MCL1. DYRK2 depletion increased FBXW7 and MCL1 levels, being the levels of MCL1 slightly lower than those obtained after FBXW7 inhibition. Interestingly, MCL1 protein levels were reduced in response to DYRK2 expression under low FBXW7 levels. These results indicate the existence of a regulatory mechanism on MCL1 accumulation that is mediated by DYRK2 but independent of FBXW7. Interestingly, DYRK2 ablation induced resistance to the anti-mitotic chemotherapy Paclitaxel in HCT116 cells (Fig. 7E), without an effect on HCT116 cell proliferation in untreated conditions (SFig. 4E). Notably, this effect was recovered in part when FBXW7 was depleted. Moreover, DYRK2 knockdown also makes HT-29 cells, a colorectal cancer cell line that harbor a wild-type FBXW7 allele, more resistant to Paclitaxel-induced cell death whereas SW837 cells, which harbor a somatic deletion of FBXW7 (L403fs) were largely resistant to Paclitaxel regardless of DYRK2 depletion (SFig. 4F). Thus, DYRK2/FBXW7 axis modulates Paclitaxel sensitivity in colorectal cancer cell lines.

Taken together, these findings demonstrate that DYRK2 participates in the degradation of multiple FBXW7 substrates and reveals DYRK2 as a general modulator of FBXW7 activity with potential consequences in the damage response and tumor progression.

## Discussion

FBXW7 is a central tumor suppressor governing cell cycle progression, cell growth, and tumor development by targeting several oncoproteins for ubiquitin-mediated proteolysis [48]. The activity and protein levels of FBXW7 are tightly controlled in different cellular contexts by protein kinases, including ERK (Extracellular Signal-Regulated Kinase), PKC (Protein Kinase C), SGK1 (Serum/Glucocorticoid Regulated Kinase 1), PLK (Polo-Like Kinases) 1 and 2, ATM and CDK5 (Cyclin Dependent Kinase 5) [42, 43, 49–52]. The manipulation of these kinases is considered a useful strategy to modulate FBXW7-dependent cell signaling pathways and tumor suppressor functions [21, 22]. Here, we identify DYRK2 as a novel FBXW7 regulatory kinase, opening new pathways for FBXW7 modulation.

Our results identified up to a total of 19 possible FBXW7 phosphorylation sites by DYRK2. Five of these sites are located within the specific N-terminus of FBXW7α. Previous studies have shown that changes in stability and location occur when this region is phosphorylated [42, 43, 51–53]; however, the effect of DYRK2 on FBXW7 is common to all isoforms (which differ only in the N-terminus), and therefore these phosphosites should not be exclusively responsible for DYRK2-dependent regulation. Similarly, phosphorylation at T205 has been described as responsible for promoting FBXW7 degradation through autoubiquitination [44, 49]. Our results rule out the involvement of SCF^FBXW7^ E3 ligase activity on the DYRK2-mediated effect, pointing to another E3 ligase/s. In this regard, some E3 ligases, as Parkin, TRIP12 or TRIM25, have been shown to target FBXW7 for proteasomal degradation [54–57]; however, none of them is able to act on the three isoforms as observed in our results. In addition, the fact that a mutant FBXW7 lacking the F-box is targeted by DYRK2 suggests that association with the SCF complex is not required. Finally, residues near or within the WD40 domain have also been identified, suggesting a possible regulation of WD40 domain functions such as substrate recognition and recruitment at the NHEJ (Non-Homologous end-joining) site after DNA damage [53]. The need for FBXW7 multisite phosphorylation to promote its destabilization has been described [42, 43, 51, 52], and this could be also the case for the destabilization of FBXW7 by DYRK2.

One of the most singular results of this work is the possible dual role of DYRK2, promoting both FBXW7 destabilization and the degradation of FBXW7 targets. In this sense, FBXW7 functional outcomes through dual regulatory pathways are common. For example, the deubiquitinase USP28 antagonizes FBXW7 autocatalytic ubiquitination and stabilizes both FBXW7 and its substrates [58]. Our results show how DYRK2 and FBXW7 activities converge to regulate common substrates implicated in relevant oncogenic signaling pathways. DYRK2 phosphorylates residues within the FBXW7 phosphodegron of c-Myc, c-Jun, mTOR, and Notch1-IC [26, 27, 33]. Therefore, the role of DYRK2 would be to create the interacting surface in the substrates for FBXW7 recruitment. On the contrary, c-Myc, c-Jun and Notch1-IC may be regulated by non-exclusive FBXW7 but DYRK2-dependent pathways, since these three transcription factors are degraded in response to DYRK2 in the absence of FBXW7. Other E3 ligases might target these proteins depending on different signals and phosphorylation by DYRK2 could act as the trigger for degradation. Indeed, at least 16 E3 ligases have been identified to ubiquitinate c-Myc [59], and the E3 ligase Itch has been described to bind the Notch1 fragment TM/ICD, thereby promoting its degradation [60]. The mechanistic aspects of DYRK2 connection to other E3 ligases are yet to be defined. Noteworthy, a DYRK2 relationship with the E3 ligases SIAH2 and the EDVP complex has been already shown [61, 62], suggesting a wider role for DYRK2 in regulating E3 ligase activities. Finally, the fact that the FBXW7-mediated degradation of Cyclin E1 is DYRK2 independent suggests the existence of subsets of FBXW7 targets with differential responses to DYRK2. Nevertheless, the dual role of DYRK2 in promoting both FBXW7 destabilization and the degradation of FBXW7 targets could be interpreted as a safety switch to limit the extent of FBWX7 activity. In the same line, DYRK2 might also trigger the degradation of these substrates through pathways independent of FBXW7. Further research is needed for a complete understanding of how DYRK2 and FBXW7 control signaling pathways via target choice and how its crosstalk is regulated.

Finally, previous studies have broadly shown that FBWX7 reduced expression or inactivating mutations correlate with poor patient prognosis in multiple cancers [21, 22]. Similarly, low DYRK2 expression has generally been reported in human tumor tissues and correlated with reduced survival [63], invasiveness [26], or poor prognosis [64]. In agreement with our results, the analysis of the protein expression levels of FBXW7 and DYRK2 in different cancer types shows an inverse correlation (SFig. 5A), particularly evident in lymphoma, renal, ovarian, and skin cancer, supporting antagonistic roles for the two proteins. In the same line, the analysis of loss-of-function mutation frequency of *DYRK2* and/or *FBXW7* revealed that mutations on both genes hardly ever happen together, which indicates that these two proteins could be working in the same pathway (SFig. 5B).

**Fig. 8:**
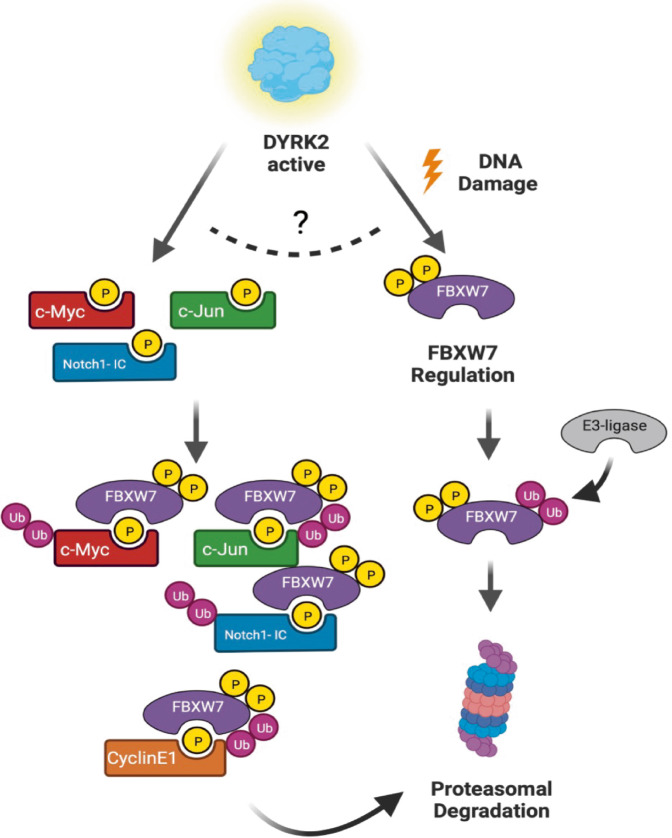
Model summarizing the results. FBXW7 stability is regulated by DYRK2.

In summary, we describe a new regulatory mechanism for FBXW7 mediated by DYRK2 (Fig. 8). On the one hand, DYRK2 participates in the FBXW7 phosphorylation promoting its degradation by the proteasome. This occurs in response to stimuli such as DNA damage, though it might not be limited to it. On the other hand, DYRK2 phosphorylation of certain FBXW7 substrates represents a necessary step for the FBXW7-dependent degradation by the proteasome of these common substrates. More research is needed to explain the balance between the two activities and which stimuli or cellular processes are involved in its control. Nevertheless, this new mechanism might have important implications for tumor development control, and points at DYRK2 as a potential target to explain the increased tumorigenesis and chemotherapy resistance caused by alterations in FBXW7.

## Supplementary information

Supplemental Material

Supplemental Figures

Supplemental Table 1

Supplemental Table 2

Supplemental Table 3

Supplemental Table 4

Original Data File

## Data Availability

All data generated or analyzed during this study are included in this published article and its additional files. Additional data are available from the corresponding author upon reasonable request.
