# Supplementary material for: FBXW7 tumor suppressor regulation by dualspecificity tyrosine-regulated kinase 2
Source: Cell Death Dis. 2023 Mar 18;14(3):202. doi: 10.1038/s41419-023-05724-0 (PMC10024693; doi:10.1038/s41419-023-05724-0)
Supplement: Supplementary file 1 — Supplemental Material [file 41419_2023_5724_MOESM1_ESM.docx]

**Supplemental Material for:**

**FBXW7 tumor suppressor regulation by dual-specificity tyrosine-regulated kinase 2.**

Rafael Jiménez-Izquierdo^1,2,3*^, Rosario Morrugares^1,2,3*^, Lucía Suanes-Cobos^1,2,3^, Alejandro Correa-Sáez^1,2,3^, Martín Garrido-Rodríguez^1,2,3^, Laura Cerero-Tejero^1,2,3^, Omar M Khan^4^, Susana de la Luna^5,6,7,8^, Rocío Sancho^9,10^ and Marco A. Calzado^1,2,3^

^1^ Instituto Maimónides de Investigación Biomédica de Córdoba (IMIBIC), Córdoba, Spain.

^2^ Departamento de Biología Celular, Fisiología e Inmunología, Universidad de Córdoba, Córdoba, Spain.

^3^ Hospital Universitario Reina Sofía, Córdoba, Spain.

^4^ Hamad Bin Khalifa University, College of Health and Life Sciences Qatar Foundation, Education City, Doha, Qatar.

^5^ Centre for Genomic Regulation (CRG), The Barcelona Institute of Science and Technology (BIST), 08003, Barcelona, Spain.

^6^ Centro de Investigación Biomédica en Red en Enfermedades Raras (CIBERER), Barcelona, Spain.

^7^ Universitat Pompeu Fabra (UPF), 08003, Barcelona, Spain.

^8^ Institució Catalana de Recerca i Estudis Avançats (ICREA), 08010, Barcelona, Spain.

^9^ Centre for Stem Cells and Regenerative Medicine, King's College London, London, SE10 9RT, UK.

^10^ Department of Medicine III, University Hospital Carl Gustav Carus, Dresden, Germany

1. **Supplemental Methods**
2. **Supplemental References**
3. **Supplemental Figure Legends**
   - Supplemental Figure 1
   - Supplemental Figure 2
   - Supplemental Figure 3
   - Supplemental Figure 4
   - Supplemental Figure 5
4. **Supplemental Tables**

- Supplemental Table 1: Reagents
- Supplemental Table 2: Mutagenesis Primers
- Supplemental Table 3: Plasmids
- Supplemental Table 4: Buffer composition

1. **Supplemental Methods**

**mRNA extraction and RT-qPCR**

Total RNA was extracted using the High Pure RNA Isolation kit (Roche Diagnostics, Mannheim, Germany), reverse transcription performed with the iScript cDNA Synthesis kit (Bio-Rad) and real-time PCR carried out in an iCYCLER detection system with iQTM SYBR Green Supermix (Bio-Rad). Amplification efficiencies were validated, and values normalized against hypoxanthine-guanine phosphoribosyltransferase (HPRT), and fold change in gene expression was calculated using the 2^−ΔΔCt^ method. Primer sequences are described in Supplemental Table 1.

***In vitro* kinase (IVK) analysis**

Commercial purified or immunoprecipitated FBXW7 and DYRK2 human recombinant proteins were incubated in kinase buffer with or without 100 µM ATP for 60 min at 37 ºC followed by WB. Buffer composition is described in Supplemental Table 4.

**Immunofluorescence**

Cells were seeded on glass coverslips and 48 h after transfection fixed with 3.7% of pre-warmed paraformaldehyde/PBS for 10 min, permeabilized with 0.1% Triton X-100/PBS for 15 min, blocked with 3% Bovine Serum Albumin (BSA)/PBS and incubated overnight with primary antibodies. After being washed with PBS and incubated for 45 min with the secondary antibody, cells were mounted on glass slides with mounting medium and 4′,6-diamidino-2-phenylindole (DAPI). Fluorescence images were captured using an LSM 5 EXCITER (Carl Zeiss MicroImaging GmbH) confocal laser scanning microscope using a 40×/1.30 oil objective (EC Plan-Neofluar) and ZEN 2008 software (Carl Zeiss MicroImaging GmbH). Images were analyzed using the ImageJ v 1.45 software (http://rsbweb.nih.gov/ij/). The degree of channel colocalization was analysed by considering the following indexes: thresholded Manders’ coefficient A and B and Pearson’s coefficient. RGB Profiler from ImageJ was used to create a profile of fluorescence intensity values across a line drawn on the image.

**Cell Viability Assays**

Cell viability of HCT116 and HT-29 cells was evaluated by MTT assay. In general, 4×10^5^ HCT116 cells or 10^6^ HT-29 cells were seeded in each well of 6-well plates. 24 h after transfection, cells were re-seeded into a 96-well plate. When cells were settled, 100 nM of Paclitaxel was added to the respective well. 72 h later, 50 μl of 3-MTT (4,5-Dimethylthiazol-2-yl)-2,5-diphenyltetrazolium bromide) (5 mg/ml) from a mixture solution of MTT:Medium (1:2) per well was added and cells were incubated for 4 h at 37 °C in darkness. Then, supernatant was removed and 100 μl DMSO was added to each well for 10 min, in gentle shaking. Absorbance was measured at 550 nm. Cell viability of Jurkat and MOLT-4 cells was evaluated by XTT assay. 48 hours followed cells transfection, cells were subcultured in a density of 10^4^ cells per well of 96-well plate. BET inhibitors were then added at the specific conditions and incubated for at least 48 hours. XTT solution was prepared dissolving XTT (10194032, Fisher Scientific) at a final concentration of 1 mg/ml in culture medium. To create the detection solution, 2.5 μl of 0.01 M Phenazine methosulfate (10626332, Fisher Scientific) was added to 1 ml of the XTT solution. Immediately, 50 μl of the detection solution was added to each well and incubated for 4 hours in darkness. Finally, absorbance at 450 nm was measured. All viability assays were measured using a TriStar LB 941 (Berthold Technologies, GmbH & Co. KG). All experiments were measured in triplicate wells and the mean values were considered for each independent experiment.

**Protein structure modelling**

The 3D FBXW7 structure model was generated with the I-TASSER server (https://zhanglab.ccmb.med.umich.edu/I-TASSER/) [1] using the FBXW7 amino acid sequence Q969H0-1 from UniProt (www.uniprot.org), and its partial crystal structure 2OVP from the Protein Data Bank (PDB; www.rscb.org). The different predictive models were classified according to their values in 3 parameters commonly used to determine the closeness between the generated model and the structure adopted by the native protein (C-score, TM-score, and the root of the error Middle Quadratic), and we choose the best model according to these parameters. Molecular graphics and analyses were performed with ChimeraX, developed by the Resource for Biocomputing, Visualization, and Informatics at the University of California, San Francisco, with support from National Institutes of Health R01-GM129325 and the Office of Cyber Infrastructure and Computational Biology, National Institute of Allergy and Infectious Diseases [2].

**Mass spectrometry (MS) analysis**

**Sample preparation:** Protein samples from IVK assays were cleaned by protein precipitation with trichloroacetic acid (TCA)/acetone and solubilized in 50 μl of 0.2% RapiGest (Waters, Milford, MA, USA) in 50 mM ammonium bicarbonate. Total protein was quantified using Qubit Protein Assay Kit (Thermo Fisher Scientific, Waltham, Massachusetts, USA). Protein samples (50 μg) were incubated with 5 mM DTT at 60 °C for 30 min, and then with 10 mM iodoacetamide at room temperature for 30 min in darkness. Sequencing Grade Modified Trypsin (Promega, Madison, Wisconsin, USA) was added (ratio 1:40 trypsin:protein) and samples were incubated at 37 °C for 2 h. Trypsin was added again (ratio 1:40) and samples were incubated at 37 °C for 15 h. RapiGest was suppressed by precipitation with 0.5% trifluoroacetic acid (TFA) at 37 °C for 1 h and centrifugation. The final volume was adjusted with milliQ water and acetonitrile (ACN) to a final concentration of 0.5 μg peptide/μl (2.25% ACN and 0.2% TFA), and 1× of the iRT peptides (Biognosis AG, Schlieren/Zurich, Switzerland) were spiked in each sample.

**LC-MS analysis**: Samples were analyzed using an LTQ-Orbitrap Fusion Lumos mass spectrometer (Thermo Fisher Scientific, San Jose, California, USA) coupled to an EASY-nLC 1 000 (Thermo Fisher Scientific (Proxeon), Odense, Denmark). Peptides were loaded directly onto the analytical column and were separated by reversed-phase chromatography using a 50-cm column with an inner diameter of 75 μm, packed with 2 μm C18 particles spectrometer (Thermo Scientific, San Jose, California, USA). Chromatographic gradients started at 95% buffer A and 5% buffer B with a flow rate of 300 nl/min for 5 min and gradually increased to 25% buffer B and 75% buffer A in 52 min and then to 40% buffer B and 60% buffer A in 8 min. After each analysis, the column was washed with 10% buffer A and 90% buffer B for 10 min. The mass spectrometer was operated in positive ionization mode with nanospray voltage set at 2.4 kV and source temperature at 275 °C. Ultramark 1621 was used for external calibration of the FT mass analyzer prior the analyses, and an internal calibration was performed using the background polysiloxane ion signal at m/z 445.1200. The acquisition was performed in data-dependent acquisition mode and full MS scans with 1 micro scan at a resolution of 120 000 were used over a mass range of m/z 350-1,500 with detection in the Orbitrap mass analyzer. Auto gain control was set to 1E5 and charge state filtering disqualifying singly charged peptides was activated. In each cycle of data-dependent acquisition analysis, following each survey scan, the most intense ions above a threshold ion count of 10 000 were selected for fragmentation. The number of selected precursor ions for fragmentation was determined by the “Top Speed” acquisition algorithm and a dynamic exclusion of 60 s. Fragment ion spectra were produced via high-energy collision dissociation at normalized collision energy of 28% and they were acquired in the ion trap mass analyzer. AGC was set to 1E4, and an isolation window of 1.6 m/z and a maximum injection time of 200 ms was used. Digested BSA (New England Biolabs cat # P8108S) was analyzed between each sample to avoid sample carryover and to assure stability of the instrument and QCloud [3] was used to control instrument longitudinal performance.

**Data analysis:** The spectra acquired were analyzed with the Proteome Discoverer software suite (v2.3, Thermo Fisher Scientific) and the Mascot search engine (v2.6, Matrix Science) [4]. The data was used to search the SwissProt human database (February 2020), including a list of common contaminants and the corresponding decoy entries [5]. For peptide identification, a precursor ion mass tolerance of 7 ppm was used for MS1, with trypsin as the chosen enzyme and up to three miscleavages allowed. The fragment ion mass tolerance was set to 0.5 Da for MS2. Oxidation of methionine and N-terminal protein acetylation were used as variable modifications, whereas carbamidomethylation on cysteine was set as a fixed modification. In the analysis of phosphorylated peptides, phosphorylation of serine, threonine and tyrosine were also set as variable modification. False discovery rate was set to a maximum of 5% in peptide identification. Phosphosite v6.6.0.2 (https://www.phosphosite.org) [6] was used for collecting information of FBXW7 phosphosites.

1. **Supplemental References**

1 Yang J, Zhang Y. I-TASSER server: new development for protein structure and function predictions. *Nucleic Acids Res* 2015; 43: W174-181.

2 Goddard TD, Huang CC, Meng EC, Pettersen EF, Couch GS, Morris JH *et al*. UCSF ChimeraX: Meeting modern challenges in visualization and analysis. *Protein Sci* 2018; 27: 14-25.

3 Chiva C, Olivella R, Borras E, Espadas G, Pastor O, Sole A *et al*. QCloud: A cloud-based quality control system for mass spectrometry-based proteomics laboratories. *PLoS One* 2018; 13: e0189209.

4 Perkins DN, Pappin DJ, Creasy DM, Cottrell JS. Probability-based protein identification by searching sequence databases using mass spectrometry data. *Electrophoresis* 1999; 20: 3551-3567.

5 Beer LA, Liu P, Ky B, Barnhart KT, Speicher DW. Efficient Quantitative Comparisons of Plasma Proteomes Using Label-Free Analysis with MaxQuant. *Methods Mol Biol* 2017; 1619: 339-352.

6 Hornbeck PV, Zhang B, Murray B, Kornhauser JM, Latham V, Skrzypek E. PhosphoSitePlus, 2014: mutations, PTMs and recalibrations. *Nucleic Acids Res* 2015; 43: D512-520.

7 Lara-Chica M, Correa-Saez A, Jimenez-Izquierdo R, Garrido-Rodriguez M, Ponce FJ, Moreno R *et al*. A novel CDC25A/DYRK2 regulatory switch modulates cell cycle and survival. *Cell Death Differ* 2022; 29: 105-117.

1. **Supplemental Figure Legends**

**Supp. Figure 1. DYRK2** **modulates FBXW7 protein levels.** (**A**) HEK-293T cells expressing Flag-FBXW7α, Flag-DYRK2 WT and Flag-DYRK2 KD were analyzed by WB using the indicated antibodies. (**B**) HEK-293T cells were transfected to express Myc-DYRK2 and endogenous protein expression for the indicated F-box family members was evaluated by WB with specific antibodies. (**C**) HEK-293T cells were transfected to express the different DYRK family members in combination with Flag-FBXW7α plasmid. Protein expression was evaluated by WB with the indicated antibodies. (**D, E**) HEK-293T cells were transfected with the indicated plasmids and after 36 h, treated with the protein synthesis inhibitor cycloheximide (CHX; 100 μg/ml) for 4, 8, 12 and 24 h. Cell lysates were analyzed by WB. The graph represents the mean ± SD of band intensity from 3 different experiments. ***P < 0.001. (**F**) HEK-293T cells expressing HA-FBXW7α, a full F-Box domain deletion mutant (Full-ΔFBox, Δ 285-324 aa) and Flag-DYRK2 WT were analyzed by WB using the indicated antibodies. (**G**) HEK-293T cells were transfected to express DYRK2 WT and/or a dominant-negative version of Cullin-1 (DN-Cul1) and protein expression was evaluated by WB with the indicated antibodies. (**H**) Transfected HEK-293T cells with the indicated plasmids were treated with MG-132 (10 μM) for 12 h. Ubiquitinated proteins were purified by anti-HA immunoprecipitation and FBXW7α detected by WB. Note: a representative experiment is shown in each panel of 3-4 performed.

**Supp. Figure 2. DYRK2 phosphorylates FBXW7.** (**A**) Flag-FBXW7α expressed in HEK-293T cells was purified by anti-Flag immunoprecipitation and used in IVK assays in the presence of purified DYRK2 protein. DYRK2 inhibitor LDN192960 was employed at 10 μM in the IVK reactions. DYRK2 mobility shift in the presence of ATP is due to autophosphorylation [7]. (**B**) FBXW7α protein sequence showing the peptides identified in the MS analysis, boxed in grey, with the phosphorylated residues labeled in red (underlined and bold amino acid are residues identified as phosphorylated at 100% probability level). (**C**) Representative spectra for the phosphorylated peptides identified in FBXW7α. (**D**) Multiple sequence alignment of FBXW7 proteins performed with Clustal Omega (https://www.ebi.ac.uk/Tools/msa/clustalo/), using default settings. The protein sequences are as follows: *Homo sapiens* isoform α or isoform 1 (NP_361014.1); *Mus musculus* isoform α or isoform 1 (NP_001171244.1); *Bos taurus* (NP_001069717.1); *Gallus gallus* (NP_001336651.1); *Xenopus laevis* isoform X1 (XP_018098751.1). Boxes show protein domains following the color code of Figure 4B. The amino acids phosphorylated by DYRK2 are shown in red (the asterisk marks those included in the FBXW7-6A mutant). In blue, are amino acids not found in the phosphoproteomic screen but included in the analysis shown in Figure S2G. (**E**) Cartoon-atom model of FBXW7α 3D structure prediction to reveal the position of buried residues (in red) located within the WD40 domain. The color code is indicated in Figure 4B. (**F**) Extracts from HEK-293T cells transfected with Flag-FBXW7α WT or the indicated mutants alone or together with Flag-DYRK2 WT were analyzed by WB. (**G**) Extracts from HEK-293T cells transfected with Flag-FBXW7α WT or the indicated mutants alone or together with Flag-DYRK2 WT were analyzed by WB. (**H**) HEK-293T cells expressing Flag-FBXW7 WT and Flag-FBXW7 6A mutant with or without Myc-DYRK2 were used in immunoprecipitation experiments with a Myc antibody. Cells were treated with MG-132 (10 μM) to avoid FBXW7 degradation. Both the lysate (5%) (INPUT) and the immunoprecipitates (IP) were analyzed by WB. General comment: a representative experiment is shown in each panel of 3-4 performed.

**Supp. Figure 3. DYRK2 and FBXW7 colocalizes in response to DNA damage.** (**A**) CHO cells were transfected with GFP-DYRK2 and Flag-FBXW7α and analyzed for the subcellular localization by immunofluorescence followed by confocal microscopy in cells treated with ETP (10 μM) or vehicle for 6 h and with MG-132 (10 μM) for the last 4 h to promote FBXW7 stabilization. DNA was stained with DAPI. Overlapping localization is shown in yellow. Fluorescence intensity profiles through the white line shown in the “RGB profile” panels indicate GFP-DYRK2 and Flag-FBXW7 cellular localization in both control and DNA damage conditions. Pearson’s coefficient (0.477) and thresholded Manders’ coefficients A and B (A = 0.9539; B = 0.8714) were calculated.

**Supp. Figure 4. DYRK2 modulates FBXW7 activity with implications on chemotherapy resistance.** (**A**) FBXW7 phospho-degron motif in the indicated proteins with the DYRK2 phosphorylation sites shown in red. (**B**) HCT116 cells WT (+/+) and knock-out (-/-) for FBXW7 were transfected with increasing concentrations of Flag-DYRK2 plasmid. Protein expression of endogenous FBXW7α, DYRK2, Notch1-IC and Cyclin E1 were evaluated by WB. (**C**) HeLa cells were treated for 12 h with DOX (1.5 μg/mL) and MG-132 (10 μM). DYRK2 was immunoprecipitated and the presence of endogenous FBXW7α in the immunoprecipitates was analyzed by WB**.** (**D**) Jurkat or MOLT4 cells were transfected with Flag-DYRK2 together with a GFP control expression vector. After 36 h cell extracts were analyzed by WB with the indicated antibodies (left panel). Transfection efficiency in these cell lines was assessed by GFP detection (right panel). Representative images of transfected Jurkat and MOLT4 cells (GFP+). Quantitative analysis of the percentage of GFP+ cells among the total population. (**E**) Cell proliferation of HCT116 cells of indicated conditions, as estimated by cell counting on indicated days (mean ± SD, n = 3). (**F**) Viability of colorectal cancer cell lines with the indicated genotypes, treated with Paclitaxel (100 nM) for 72 h as measured by MTT assay and shown relative to control-treated cells (mean ± SD, n = 3, *P < 0.05, n.s., not significant).

**Supp. Figure 5. Analysis of the expression levels and mutations of FBXW7 and DYRK2 in different cancer types** (**A**) DYRK2 and FBXW7 protein abundance in tumor tissues obtained from The Human Protein Atlas. Column and circle color shows the antibody stain levels observed in tumor tissues. The point size indicates the number of patients showing expression relative to the total patients. The tumor tissues were sorted based on the abundance score differences between proteins. To calculate this, every staining level was assigned to a number (Not detected: 1, Low: 2, Medium: 3 and High: 4) and multiplied by the number of patients for each tissue and protein. Then, the absolute mean differences were calculated for every tumor tissue. (**B**) *DYRK2* and *FBXW7* mutation frequency (missense, non-sense, or deep deletions) for each gene or together for every tumor type included in the TCGA PanCancer dataset.

**Supplemental Table 1: Reagents.** Reagents used in this paper.

**Supplemental Table 2: Primers. Mutagenesis** primers used in this paper.

**Supplemental Table 3: Plasmids**. Plasmids used in this paper.

**Supplemental Table 4: Buffer composition**. Buffer composition used in this paper.
