# Supplementary material for: FBXW7 tumor suppressor regulation by dualspecificity tyrosine-regulated kinase 2
Source: Cell Death Dis. 2023 Mar 18;14(3):202. doi: 10.1038/s41419-023-05724-0 (PMC10024693; doi:10.1038/s41419-023-05724-0)

Supplemental Figure 1

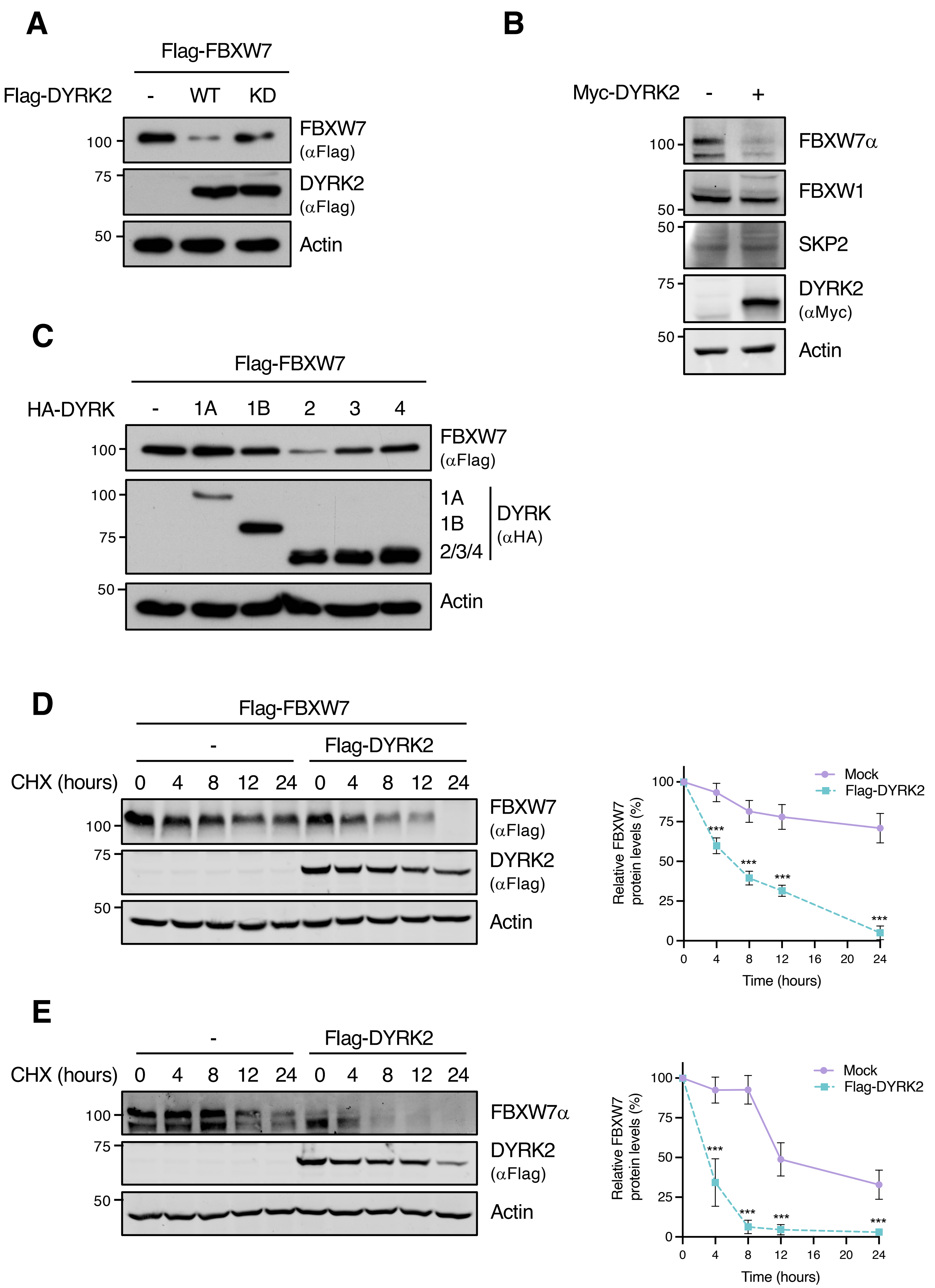

Supplemental Figure 1

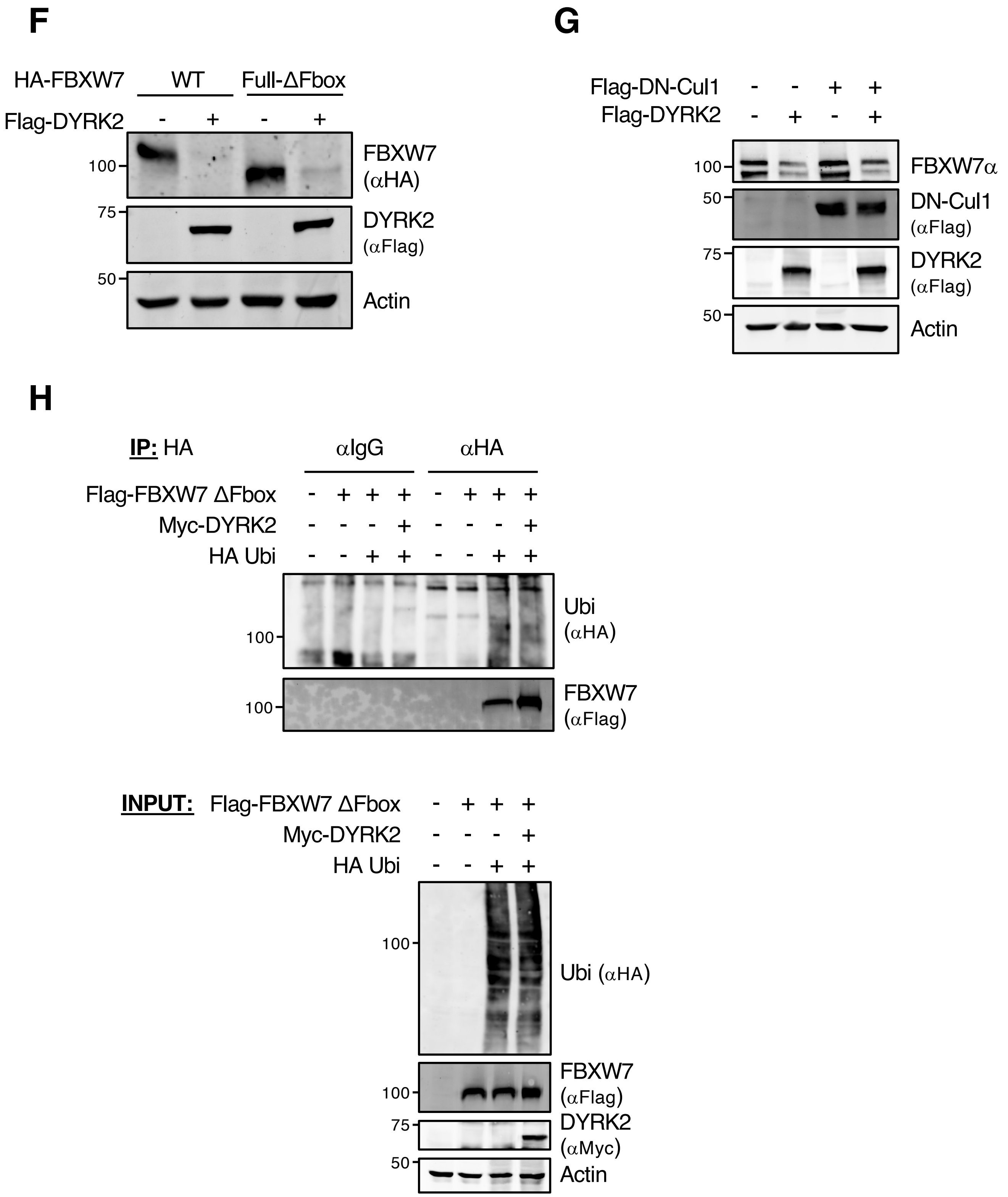

Supplemental Figure 2

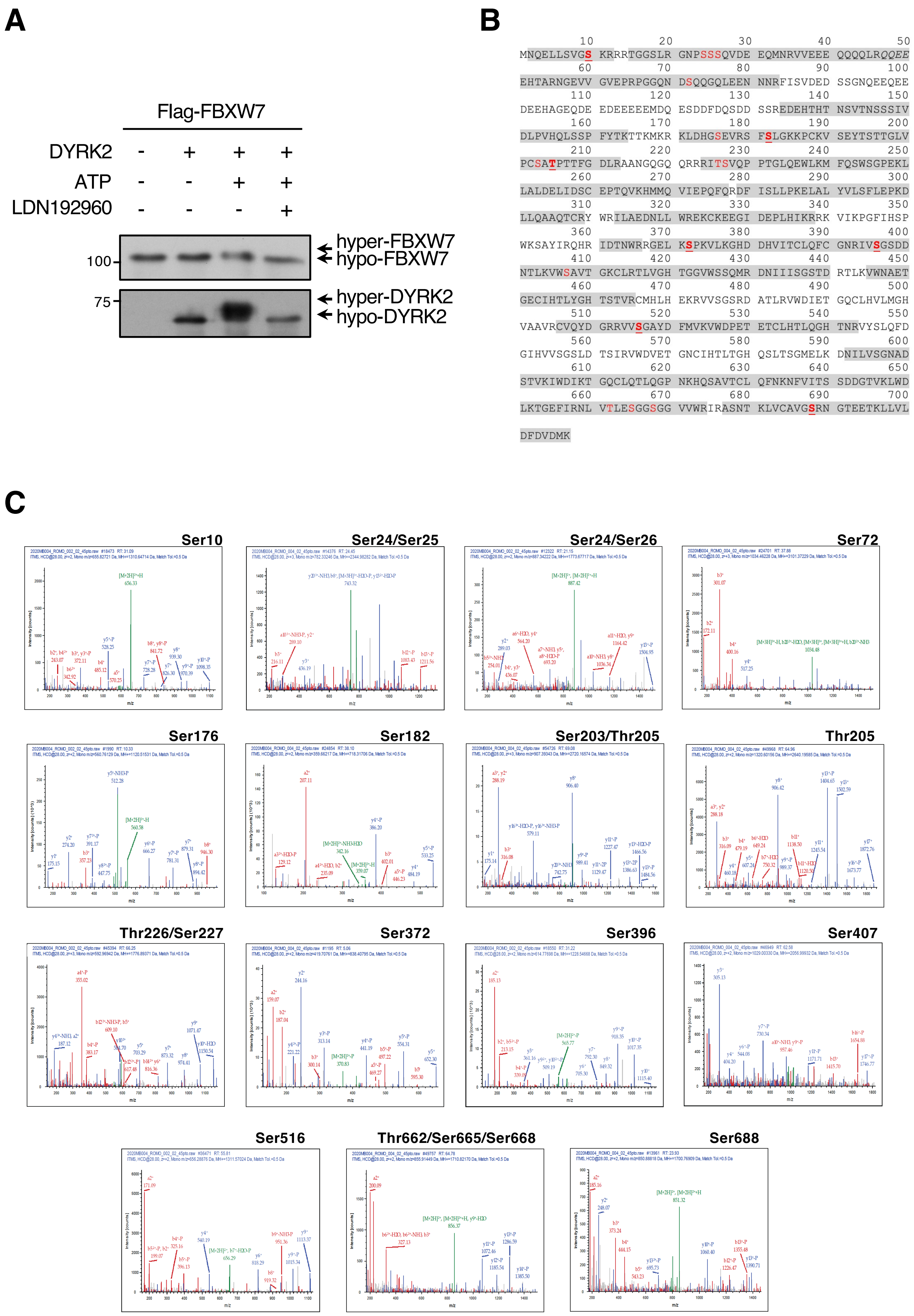

Supplemental Figure 2

D

```
                S10      S18 S24/25/26
H. sapiens     MNQELLSVGSKRRRTGGSLRGNPSSSQVDEEQMNRVVEEEQQQQLRQQEEEHTARNGEVV   60
M. musculus    MNQELLSVGSKRRRTGGSLRGNASSSQVDEGQMNRVVEEDPQQQARHQEEEHTARNGELV   60
B. taurus      ------------------------------------------------------------    0
G. gallus      MNQELLSVGSKRRRTGGSLRANASSSQVDEEQMNRVVEEEQQQRQQQEEQHIGRNGEIR   60
X. laevis      MNQELLSVGSKRRRTGGSVRGNASSSQADEEQMNRVVEEEEQQ-----QEEHIVLNGEID   55

                    S72
H. sapiens     GVEPRPGGQNDSQQGQLEENNNRFISVDEDSSGNQEE------QEEDEEHAGE---QDEE  111
M. musculus    GANPRPGDQNDTQQGQVEENNNRFISVDEDSSGNQEEQ------EEDEEHAGEQEEEEEE  114
B. taurus      ----------------------MCVPRSGLILSCICLYCGVLLPVLLPNLPFLTCLSMS   37
G. gallus      E-EPGFDDRRESQQEQLEENNNRLITVDEESTCNQEE--------DDDEHAGDQ---EEE  108
X. laevis      GDAPGSLERNEPHQEHLEENNNSCTIAD-QESADQPGEHELSHQDEDEDEEAC---DAED  111

H. sapiens     DEEEEEMDQESDDFDQSDDSSREDEHTHTNSVTNSSSIVDLPVHQLS-SPFYTKTTKMKR  170
M. musculus    EEEEEEMDQESDDFDPSDDSSREDEHTHNSNVTNCSSVSDLPAHQLS-SPFYTKTTKMKR  173
B. taurus      TLESVTYLPEKGLYCQRLPNSRT--H-GGTESLKGKNPEN----MGFYGTLKMIFYKMKR   90
G. gallus      VEEEEEMDQESDDFDQSDDSSREDEHANSNSVTNSSSLIDLPIHQ---SSPFYIKTKMKR  165
X. laevis      EE---EMDQDSDDFEQSDDSSREEEHRNGNDIPNSNSMVDMPINQ--QSICFNPKSKMKR  166

                S176* S182*         S203 T205*         T226/S227*
H. sapiens     KLDHGSEVRSFSLGKKPCKVSEYTSTTGLVPCSATPTTFGDLRAANGQGQQRRRITSVQP  230
M. musculus    KLDHGSEVRSFSLGKKPCKVSDYTSTTGLVPCSATPTTFGDLRAANGQGQQRRRITSVQP  233
B. taurus      KLDHGSEVRSFSLGKKPCKVSEYTSTTGLVPCSATPTTFGDLRAANGQGQQRRRITSVQP  150
G. gallus      KLDHGSEVRSFSLGKKPCKVSEYTSTTGLVPCSATPTTFGDLRAANGQGQQRRRITSVQP  225
X. laevis      KLDHGSEVRSFSLGKKPCKGSEYTSTTGLVPCSATPTTFGDLRAANGQGQQRRRITSVQP  226

                  Dimerization domain                            F-box
H. sapiens     PTGLQEWLKMFQSWSGPEKLLALDELIDSCEPTQVKHMMQVIEPQFQRDFISLLPKELAL  290
M. musculus    PTGLQEWLKMFQSWSGPEKLLALDELIDSCEPTQVKHMMQVIEPQFQRDFISLLPKELAL  293
B. taurus      PTGLQEWLKMFQSWSGPEKLLALDELIDSCEPTQVKHMMQVIEPQFQRDFISLLPKELAL  210
G. gallus      PTGLQEWLKMFQSWSGPEKLLALDELIDSCEPTQVKHMMQVIEPQFQRDFISLLPKELAL  285
X. laevis      PTGLQEWLKMFQSWSGPEKLLALDELIDICEPTQVKHMMQVIEPQFQRDFISLLPKELAL  286

                     F-box                                        S349
H. sapiens     YVLSFLEPKDLLQAAQTCRYWRILAEDNLLWREKCKEEGIDEPLHIKRRKVIKPGFIHSP  350
M. musculus    YVLSFLEPKDLLQAAQTCRYWRILAEDNLLWREKCKEEGIDEPLHIKRRKIIKPGFIHSP  353
B. taurus      YVLSFLEPKDLLQAAQTCRYWRILAEDNLLWREKCKEEGIDEPLHIKRRKVIKPGFIHSP  270
G. gallus      YVLSFLEPRDLLQAAQTCRYWRILAEDNLLWREKCKEEGIDEPLHIKRRKVIKPGFIHSP  345
X. laevis      YVLSFLDPKDLLHAAQTCRYWRILAEDNLLWREKCKDDGIDEPLHIKRRKVLKPGFTHSP  346

                                S372*          WD1     S396      S407
H. sapiens     WKSAYIRQHRIDTNWRRGELKSPKVLKGHDDHVITCLQFCGNRIVSGSDDNTLKVWSAVT  410
M. musculus    WKSAYIRQHRIDTNWRRGELKSPKVLKGHDDHVITCLQFCGNRIVSGSDDNTLKVWSAVT  413
B. taurus      WKSAYIRQHRIDTNWRRGELKSPKVLKGHDDHVITCLQFCGNRIVSGSDDNTLKVWSAVT  330
G. gallus      WKSAYIRQHRIDTNWRRGELKSPKVLKGHD-HVITCLQFCGNRIVSGSDDNTLKVWSAVT  404
X. laevis      WKSAYIRQHRIDTNWRRGELKSPKVLKGHDDHVITCLQFCGNRIVSGSDDNTLKVWSAVT  406

                  WD1              WD2                           WD3
H. sapiens     GKCLRTLVGHTGGVWSSQMRDNIIISGSTDRTLKVWNAETGECIHTLYGHTSTVRCMHLH  470
M. musculus    GKCLRTLVGHTGGVWSSQMRDNIIISGSTDRTLKVWNAETGECIHTLYGHTSTVRCMHLH  473
B. taurus      GKCLRTLVGHTGGVWSSQMRDNIIISGSTDRTLKVWNAETGECIHTLYGHTSTVRCMHLH  390
G. gallus      GKCLRTLVGHTGGVWSSQMRDNIIISGSTDRTLKVWNAETGECIHTLYGHTSTVRCMHLH  464
X. laevis      GKCLRTLVGHTGGVWSSQMRDNIIISGSTDRTLKVWNAETGECIHTLYGHTSTVRCMHLH  466

                      WD3                      WD4      S516
H. sapiens     EKRVVSGSRDATLRVWDIETGQCLHVLMGHVAAVRCVQYDGRRVVSGAYDFMVKVWDPET  530
M. musculus    EKRVVSGSRDATLRVWDIETGQCLHVLMGHVAAVRCVQYDGRRVVSGAYDFMVKVWDPET  533
B. taurus      EKRVVSGSRDATLRVWDIETGQCLHVLMGHVAAVRCVQYDGRRVVSGAYDFMVKVWDPET  450
G. gallus      EKRVVSGSRDATLRVWDIETGQCLHVLMGHVAAVRCVQYDGRRVVSGAYDFMVKVWDPET  524
X. laevis      EKRVVSGSRDATLRVWNIETGQCLHVLMGHVAAVRCVQYDGRRVVSGAYDFMVKVWDPET  526

                 WD4                WD5                          WD6
H. sapiens     ETCLHTLQGHTNRVYSLQFDGIHVVSGSLDTSIRVWDVETGNCIHTLTGHQSLTSGMELK  590
M. musculus    ETCLHTLQGHTNRVYSLQFDGIHVVSGSLDTSIRVWDVETGNCIHTLTGHQSLTSGMELK  593
B. taurus      ETCLHTLQGHTNRVYSLQFDGIHVVSGSLDTSIRVWDVETGNCIHTLTGHQSLTSGMELK  510
G. gallus      ETCLHTLQGHTNRVYSLQFDGIHVVSGSLDTSIRVWDVETGNCIHTLTGHQSLTSGMELK  584
X. laevis      ETCLHTLQGHTNRVYSLQFDGIHVVSGSLDTSIRVWDVETGNCIHTLTGHQSLTSGMELK  586

                         WD6                             WD7
H. sapiens     DNILVSGNADSTVKIWDIKTGQCLQTLQGPNKHQSAVTCLQFNKNFVITSSDDGTVKLWD  650
M. musculus    DNILVSGNADSTVKIWDIKTGQCLQTLQGPSKHQSAVTCLQFNKNFVITSSDDGTVKLWD  653
B. taurus      DNILVSGNADSTVKIWDIKTGQCLQTLQGPNKHQSAVTCLQFNKNFVITSSDDGTVKLWD  570
G. gallus      DNILVSGNADSTVKIWDIKTGQCLQTLQGPNKHQSAVTCLQFNKNFVITSSDDGTVKLWD  644
X. laevis      DNILVSGNADSTVKIWDIKTGQCLQTLQGPNKHQSAVTCLQFNKNFVITSSDDGTVKLWD  646

                 WD7   T662/S665/S668        S688*
H. sapiens     LKTGEFIRNLVTLESGGSGGVVWRIRASNTKLVCAVGSRNGTEETKLLVLDFDVDMK  707
M. musculus    LKTGEFIRNLVTLESGGSGGVVWRIRASNTKLVCAVGSRNGTEETKLLVLDFDVDMK  710
B. taurus      LKTGEFIRNLVTLESGGSGGVVWRIRASNTKLVCAVGSRNGTEETKLLVLDFDVDMK  627
G. gallus      LKTGEFIRNLVTLESGGSGGVVWRIRASNTKLVCAVGSRNGTEETKLLVLDFDVDMK  701
X. laevis      LKTGEFIRNLVTLESGGSGGVVWRIRASNTKLVCAVGSRNGTEETKLLVLDFDVDMK  703
```

Supplemental Figure 2

E

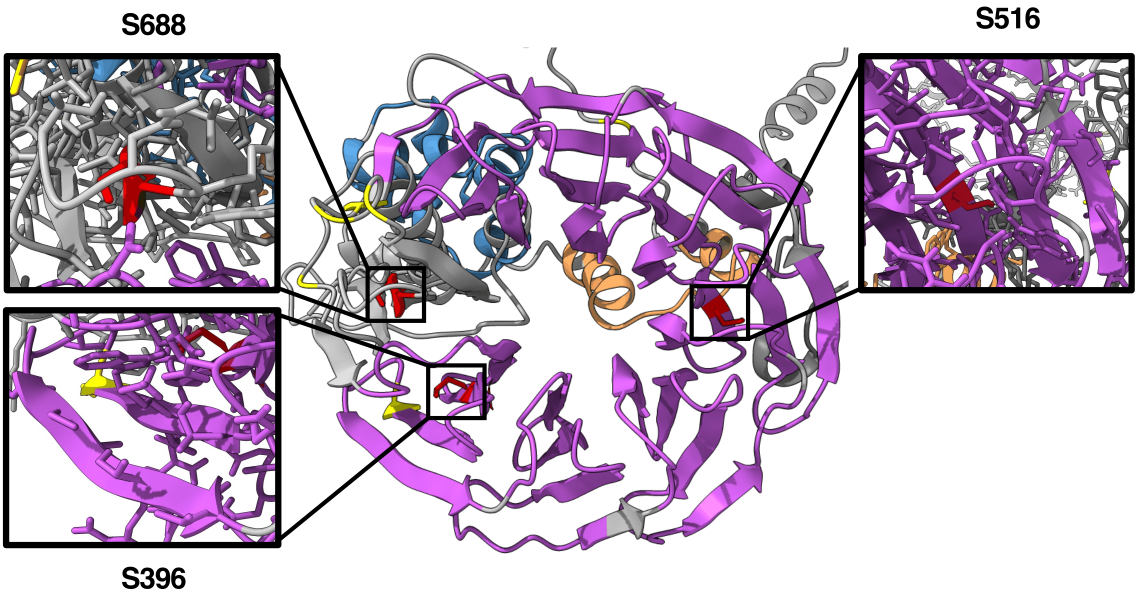

F

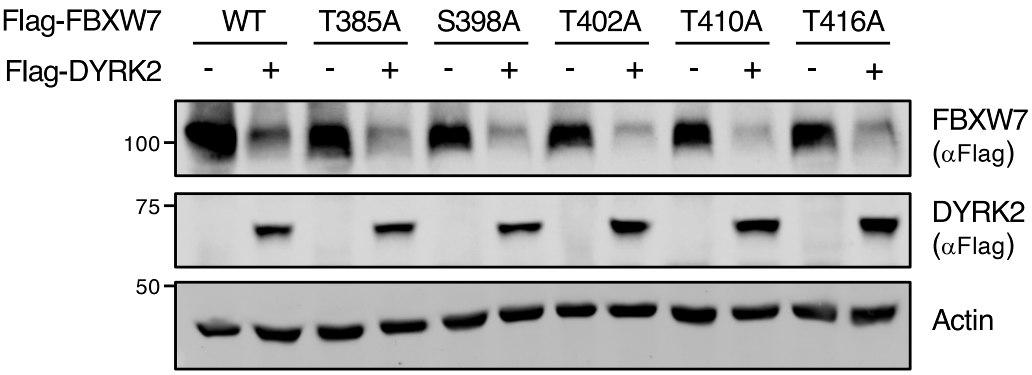

G

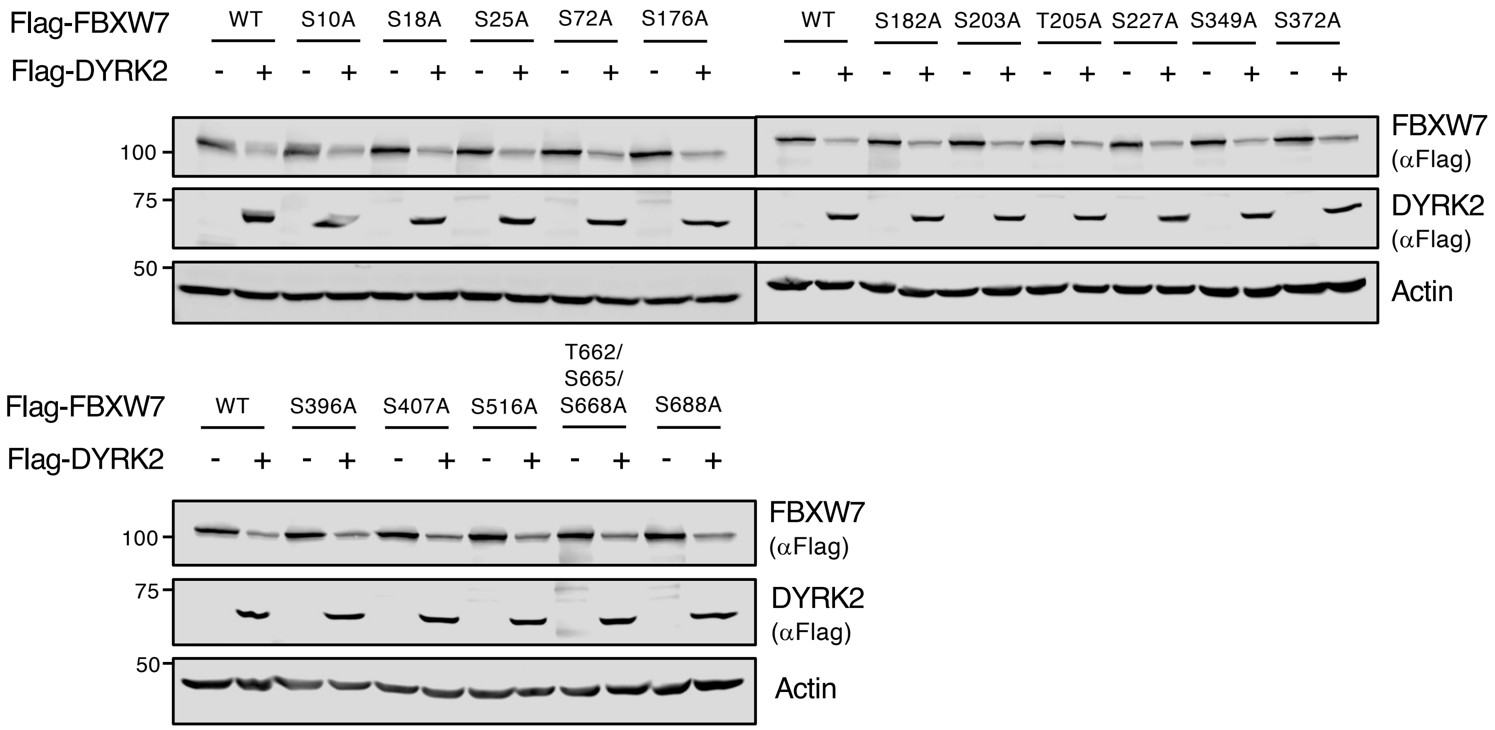

Supplemental Figure 2

H

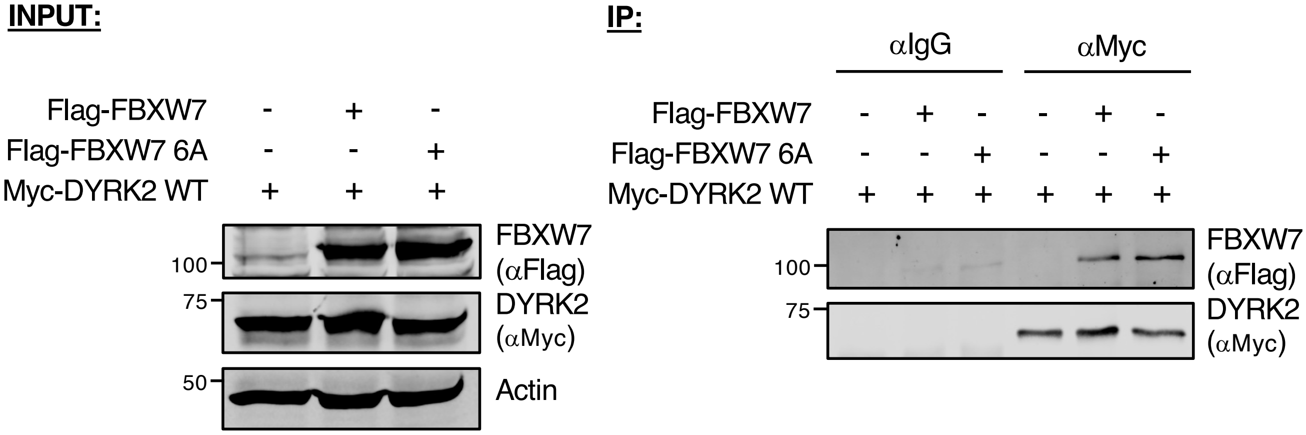

Supplemental Figure 3

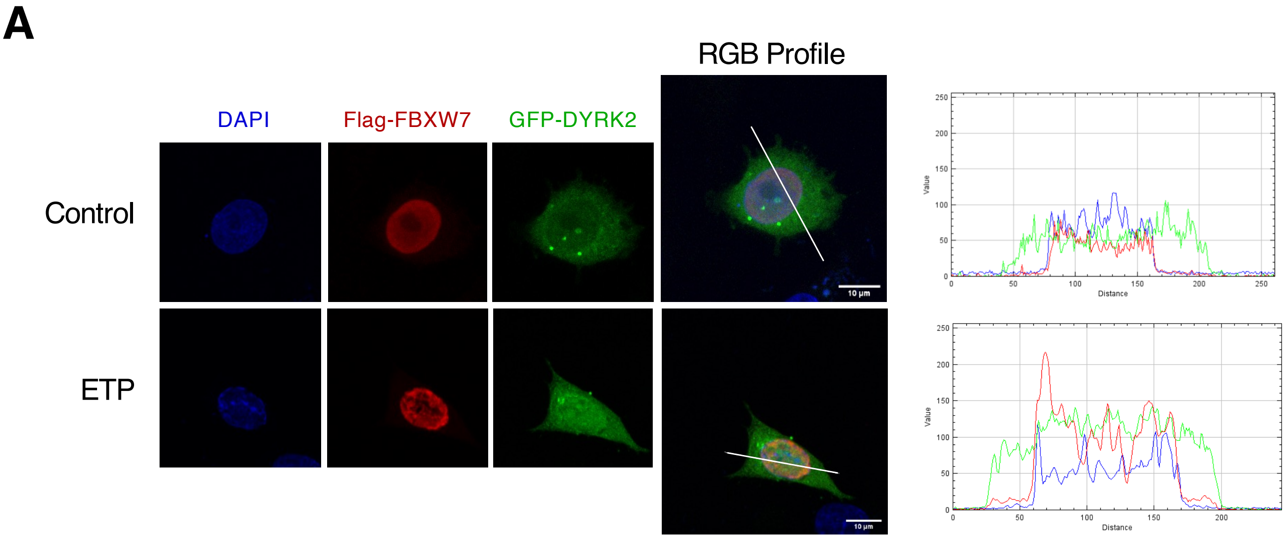

Supplemental Figure 4

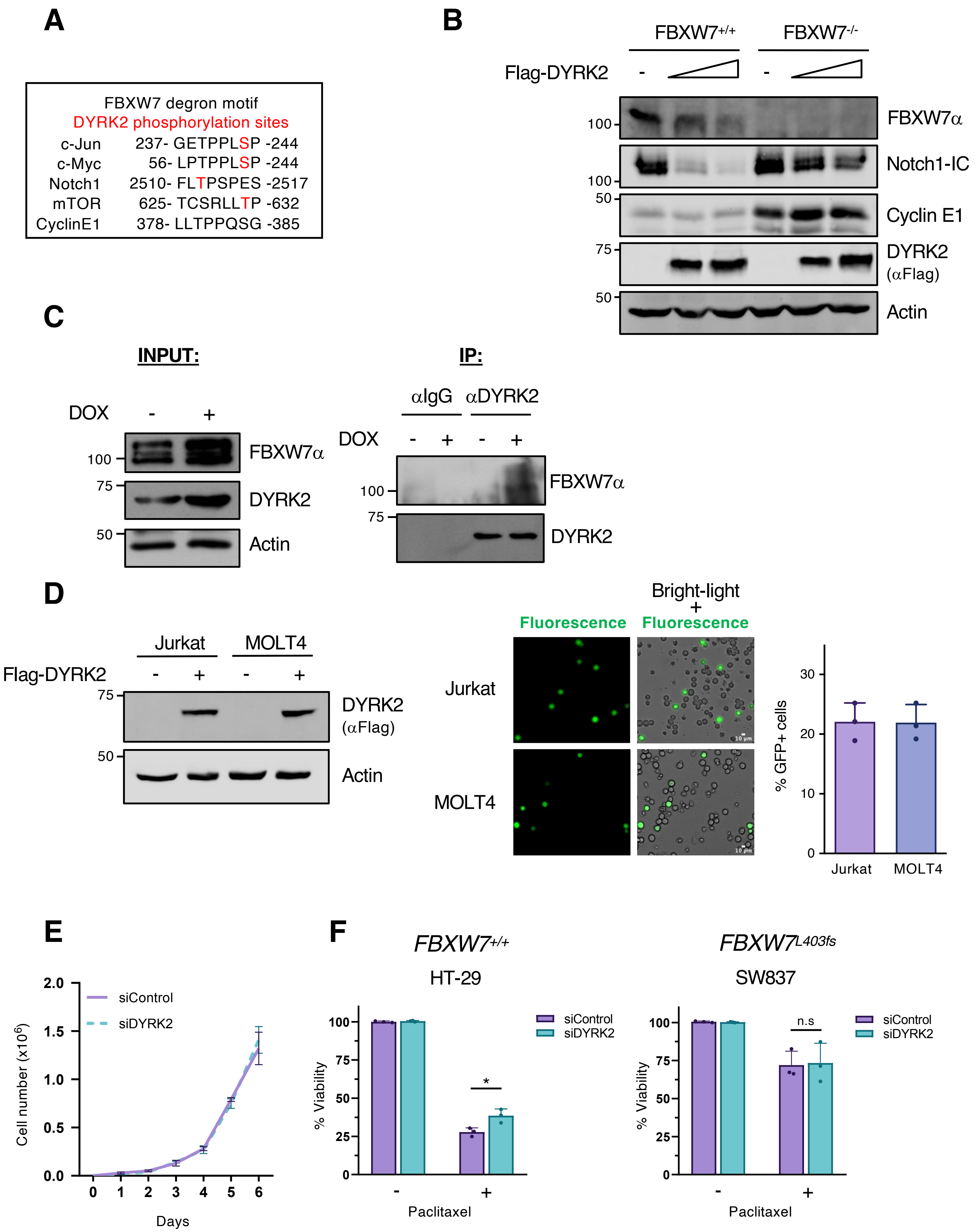

Supplemental Figure 5

A

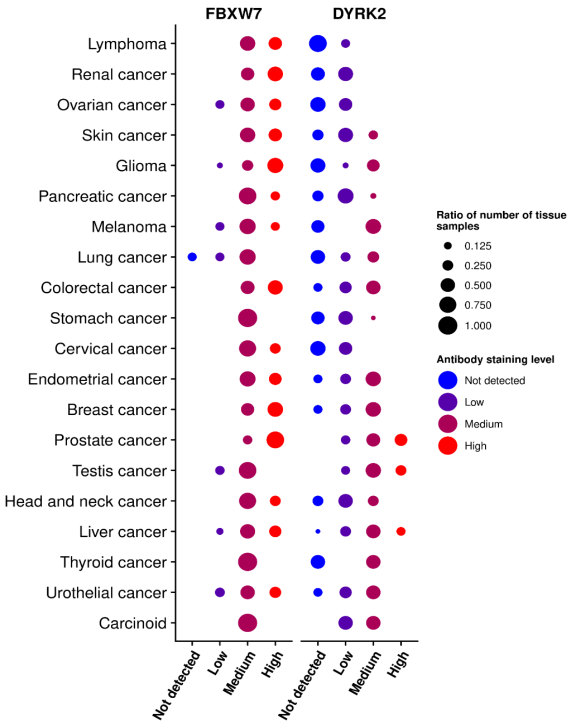

B

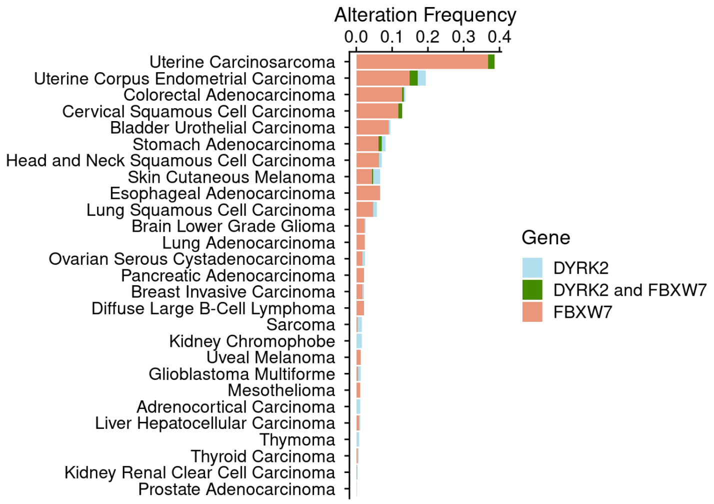

Supplement: Supplementary file 2 — Supplemental Figures [file 41419_2023_5724_MOESM2_ESM.pdf]
