# Supplementary material for: FBXW7 tumor suppressor regulation by dualspecificity tyrosine-regulated kinase 2
Source: Cell Death Dis. 2023 Mar 18;14(3):202. doi: 10.1038/s41419-023-05724-0 (PMC10024693; doi:10.1038/s41419-023-05724-0)
Supplement: Supplementary file 3 — Supplemental Table 1 [file 41419_2023_5724_MOESM3_ESM.docx]

**Supplemental Table 1**

| **REAGENT or RESOURCE** | **SOURCE** | **IDENTIFIER** |
| --- | --- | --- |
| Antibodies | | |
| Mouse monoclonal anti-FLAG M2 antibody | Sigma-Aldrich | Cat# F3165, RRID:AB_259529 |
| β-actin (AC-74) | Sigma-Aldrich | Cat# A2228, RRID:AB_476697 |
| Anti-DYRK2 polyclonal antibody | Sigma-Aldrich | Cat# HPA027230, RRID:AB_1847925 |
| HA epitope (3F10) | Roche Molecular Biochemicals | Cat# 12158167001, RRID:AB_390915 |
| GFP | Roche Molecular Biochemicals | Cat# 11814460001, RRID:AB_390913 |
| Myc (9E10) | Roche Molecular Biochemicals | Cat# 11667149001, RRID:AB_390912 |
| DYRK2 (H80) | Santa Cruz Biotechnology | Cat# sc-66867, RRID:AB_2094066 |
| p53 (DO-1) | Santa Cruz Biotechnology | Cat# sc-126, RRID:AB_628082 |
| FBXW7 | Bethyl Laboratories | Cat# A301-720A, RRID:AB_1210897 |
| DYRK2 | MRC Protein Phosphorylation and Ubiquitination Unit | Cat# S473A |
| Jun (60A8) | Cell Signaling | Cat# 9165, RRID:AB_2130165 |
| mTOR | Cell Signaling | Cat# 2972, RRID:AB_330978 |
| CyclinE1 (HE12) | Cell Signaling | Cat# 4129, RRID:AB_2071200 |
| MCL1 | Cell Signaling | Cat# 4572, RRID:AB_2281980 |
| SKP2 | Cell Signaling | Cat#4358 RRID:AB_2187660 |
| FBXW1 (β-TrCP) (D13F10) | Cell Signaling | Cat#4394, RRID: AB_10545763 |
| Notch1 | Abcam | Cat# ab52627, RRID:AB_881725 |
| Phospho-HSF1-Ser 320 | Abcam | Cat# ab76183,  RRID:AB_1523789 |
| Alexa Fluor 647 goat anti-mouse IgG1 | Thermo Fisher Scientific | Cat# A-21235, RRID:AB_2535804 |
| Alexa Fluor 488 goat anti-rabbit IgG1 | Thermo Fisher Scientific | Cat# A32731, RRID:AB_2633280 |
| Alexa Fluor 647 goat anti-rabbit IgG1 | Thermo Fisher Scientific | Cat# A32733, RRID:AB_2633282 |
| Anti-phosphoserine/threonine | ECM Biosciences | Cat# PP2551, RRID:AB_1184778 |
| HSF1 | Enzo Life Science | Cat# ADI-SPA-901-D, RRID:AB_2039202 |
| Goat anti Mouse IgG (H/L): DyLight®800 | Bio-Rad | Cat# STAR117D800GA, RRID:AB_10845157 |
| StarBright™ Blue 700 Goat Anti-Mouse IgG | Bio-Rad | Cat# 12004159, RRID:AB_2884948 |
| StarBright Blue 700 Goat Anti-Rabbit IgG | Bio-Rad | Cat# 12004161, RRID:AB_2721073 |
| StarBright™ Blue 520 Goat Anti-Rabbit IgG | Bio-Rad | Cat# 12005870, RRID:AB_2884949 |
| Rabbit F(ab')2 anti Rat IgG: Dylight®800 | Bio-Rad | Cat# STAR16D800GA, RRID:AB_10842665 |
| Bacterial and virus strains | | |
| *Escherichia coli* TOP10 | Thermo Fisher Scientific | Cat# C404010 |
| XL10-Gold Ultracompetent Cells | Agilent | Cat# 200315 |
| Chemicals, peptides, and recombinant proteins | | |
| MG-132 | Enzo Life Science | Cat# BML-PI102-0005 |
| 1NM-PP1 | Santa Cruz Biotechnology | Cat# sc-203214 |
| Protein A/G Sepharose beads | Santa Cruz Biotechnology | Cat# sc-2003, RRID:AB_10201400 |
| Paclitaxel | Sigma-Aldrich | Cat# T1912 |
| Etoposide | Sigma-Aldrich | Cat# E1383 |
| OTX015 | Cayman Chemical | Cat# 159471MG |
| XTT | Fisher Scientific | Cat# 10194032 |
| Phenazine methosulfate | Fisher Scientific | Cat# 10626332 |
| Adriamycin/Doxorubicin | Sigma-Aldrich | Cat# 44583 |
| Harmine | Sigma-Aldrich | Cat# 286044 |
| Curcumine | Sigma-Aldrich | Cat# C1386 |
| Cycloheximide | Sigma-Aldrich | Cat# C7698 |
| ATP | Sigma-Aldrich | Cat# A26209 |
| Penicillin-Streptomycin | Sigma-Aldrich | Cat# P0781 |
| cOmplete™ Protease Inhibitor Cocktail | Sigma-Aldrich | Cat# 11697498001 |
| Crystal Violet | Sigma-Aldrich | Cat# C3886 |
| Triton™ X-100 | Sigma-Aldrich | Cat# T8787 |
| Glutaraldehyde solution | Sigma-Aldrich | Cat# G6257 |
| Paraformaldehyde | Sigma-Aldrich | Cat# 8.18715 |
| Bovine Serum Albumin (BSA) | Sigma-Aldrich | Cat# A6003 |
| MTT | Sigma-Aldrich | Cat# M2128 |
| LDN192960 | MedChemExpress | Cat# HY-13455 |
| Lambda Protein Phosphatase | New England Biolabs | Cat# P0753L |
| siRNA non-targeting pool | Dharmacon | Cat# D-001810-10-20 |
| ON-TARGET plus SMARTpool against DYRK2 | Dharmacon | Cat# L-004730-00-0020 |
| ON-TARGET plus Human FBXW7 siRNA | Dharmacon | Cat# L-004264-00-0020 |
| DAPI (4',6-Diamidino-2-Phenylindole, Dihydrochloride) | Thermo Fisher Scientific | Cat# D1306, RRID:AB_2629482 |
| Polyethylenimine, Linear, MW 25000, Transfection Grade (PEI 25K™) | Polysciences | Cat# 23966 |
| ROTI®Fect | Carl Roth | Cat# P001.4 |
| Lipofectamine™ 2000 Transfection Reagent | Invitrogen™ | Cat# 10696153 |
| Dulbecco’s Modified Eagle Medium high glucose, GlutaMAX™ Supplement | Gibco | Cat# 61965026 |
| RPMI 1640 Medium, GlutaMAX™ Supplement | Gibco | Cat# 61870010 |
| Fetal Bovine Serum FBS | Gibco | Cat# 10270106 |
| Trypsin-EDTA (0.25%), phenol red | Gibco | Cat# 25200072 |
| FBXW7 recombinant protein | MyBioSource | Cat# MBS7053567 |
| DYRK2 recombinant protein | Merck | Cat# 14-669M |
| Critical commercial assays | | |
| QuikChange Lightning Site-Directed Mutagenesis Kit | Agilent | Cat# 210518 |
| Experimental models: Cell lines | | |
| HEK293T | ATCC | CRL-3216 |
| HeLa | ATCC | CCL-2 |
| HEK293T DYRK2^-/-^ | Laboratory of Laureano de la Vega | Moreno et al., 2020; Morrugares et al., 2020 |
| HeLa DYRK2^-/-^ | Laboratory of Laureano de la Vega | Moreno et al., 2020; Morrugares et al., 2020 |
| MDA-MB-468 DYRK2^-/-^ | Laboratory of Laureano de la Vega | Moreno et al., 2020; Morrugares et al., 2020 |
| MDA-MB-231 DYRK2^-/-^ | Laboratory of Laureano de la Vega | Moreno et al., 2020; Morrugares et al., 2020 |
| HCT-116 FBXW7^-/-^ | Laboratory of Professor Bert Vogelstein | PMID: 14999283 |
| CHO | ATCC | CCL-61 |
| A549 | ATCC | CCL-185 |
| Jurkat | ATCC | TIB-152 |
| MOLT-4 | ATCC | CRL-1582 |
| HCT116 | ATCC | CCL-247 |
| HT-29 | ATCC | HTB-38 |
| SW837 | ATCC | CCL-235 |
| Oligonucleotides | | |
| Human FBXW7 realtime PCR primer forward: TCTGAGGTCCGCTCTTTTTCTT | This study | |
| Human FBXW7 realtime PCR primer reverse: TGAGGTCCCCAAAAGTTGTTG | This study | |
| Human HPRT realtime PCR primer forward: AATTATGGACAGGACTGAACGTCTTGCT | This study | |
| Human HPRT realtime PCR primer forward: TCCAGCAGGTCAGCAAAGAATTTATAGC | This study | |
| See TableS2 for the list of Oligos for FBXW7 Mutagenesis | This study | |
| Software and algorithms | | |
| I-TASSER | Zhanglab | https://zhanglab.ccmb.med.umich.edu/I-TASSER/ |
| Image Lab | BioRad | https://www.bio-rad.com/ en-us/product/image-labsoftware?ID=KRE6P5E8Z |
| FireBrowse R | FireBrowse R | https://github.com/mariodeng/FirebrowseR |
| ImageJ v1.45 | ImageJ | https://imagej.nih.gov/ij/ |
| Fiji | Fiji | https://image.net/fiji |
| Prism7 | GraphPad software | https://www.graphpad.com/  scientific-software/prism/ |
| ChimeraX | ChimeraX | https://www.rbvi.ucsf.edu/chimerax/ |
| GIMP | The GIMP Team | https://www.gimp.org/ |
| Other | | |
| See TableS3 for the list of plasmids used in this paper | This study | N/A |
| See TableS4 for the list of buffers used in this paper | This study | N/A |
