# Supplementary material for: FBXW7 tumor suppressor regulation by dualspecificity tyrosine-regulated kinase 2
Source: Cell Death Dis. 2023 Mar 18;14(3):202. doi: 10.1038/s41419-023-05724-0 (PMC10024693; doi:10.1038/s41419-023-05724-0)
Supplement: Supplementary file 7 — Original Data File [file 41419_2023_5724_MOESM7_ESM.pdf]

Original Western Blot

Fig. 1A

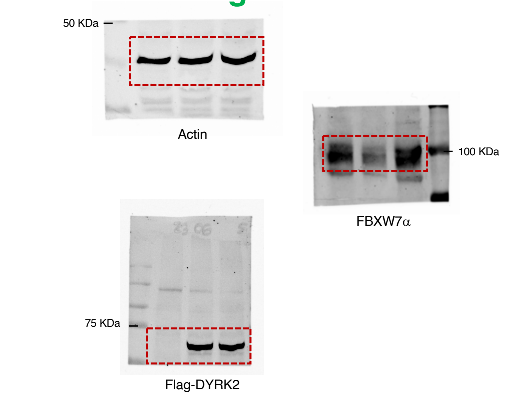

Fig. 1B

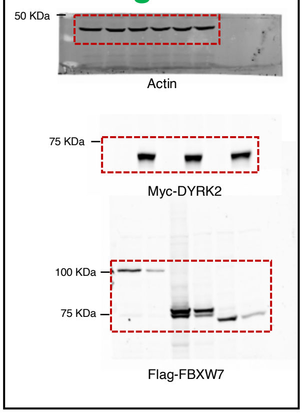

Fig. 1C

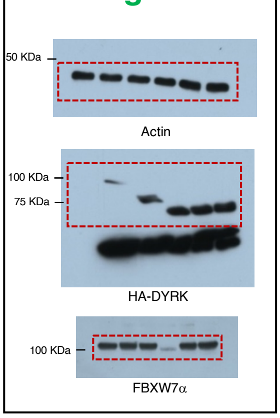

Fig. 1D

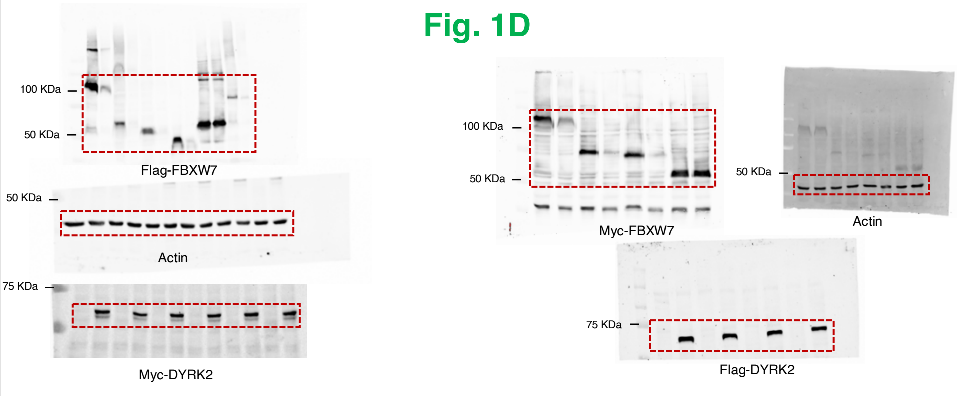

Fig. 2A

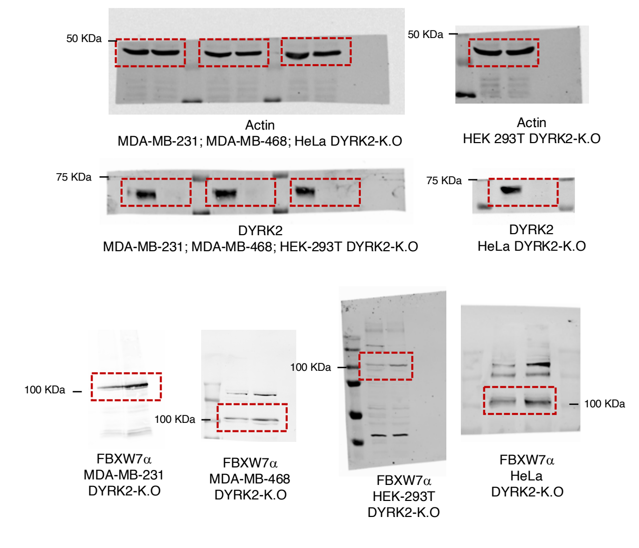

Fig. 2B

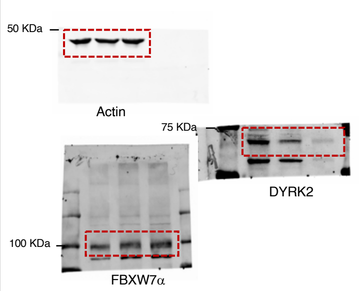

Fig. 2C

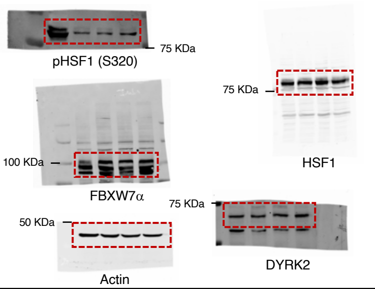

Fig. 2D

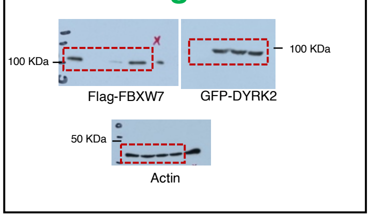

Fig. 2E

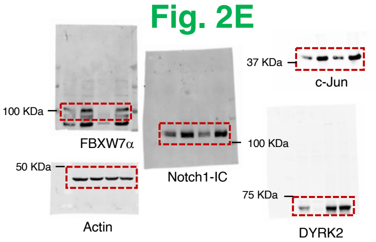

Fig. 3A

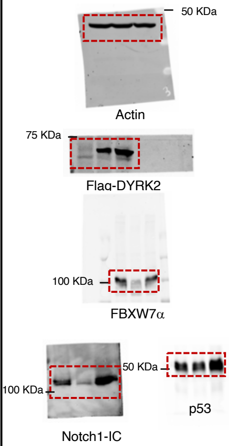

Fig. 3B

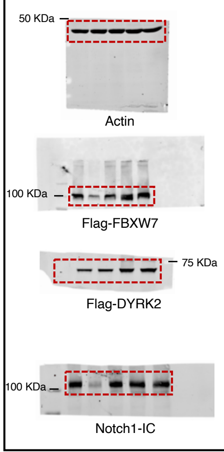

Fig. 3C

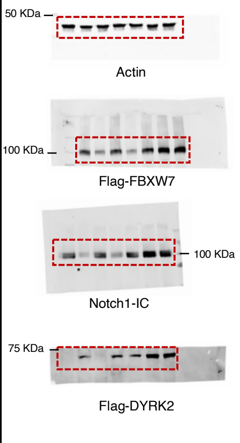

Fig. 3D

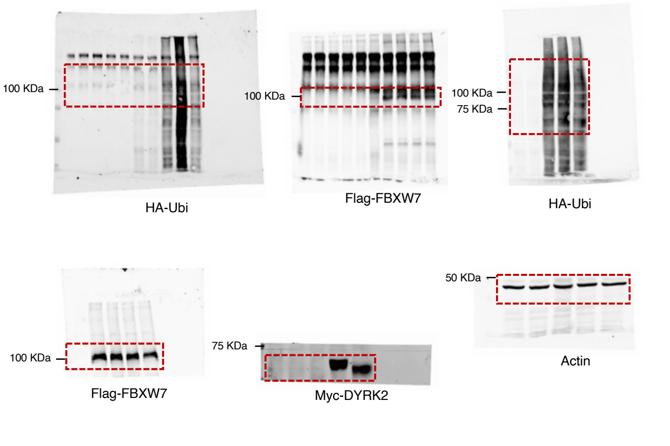

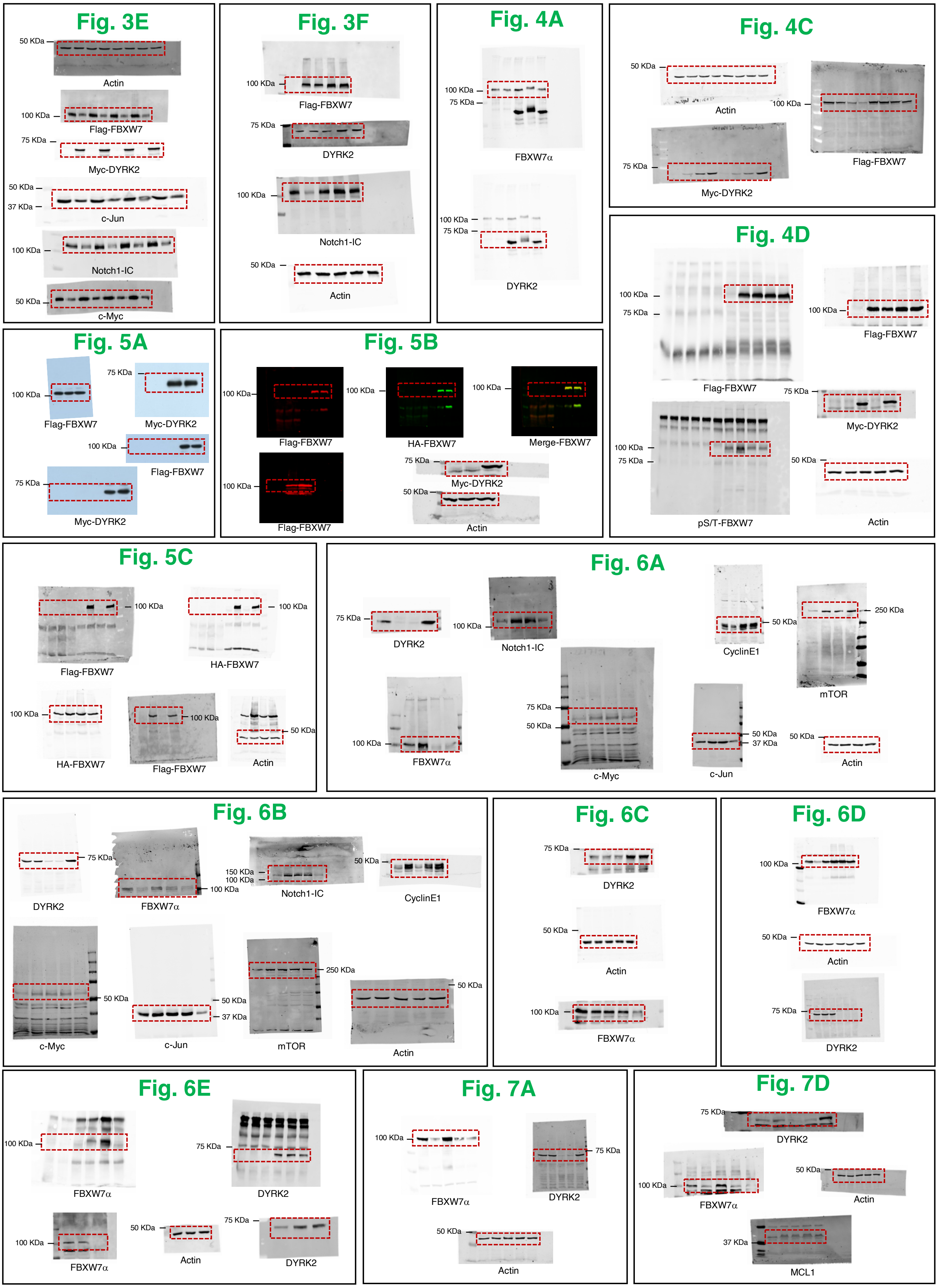
Fig. 3E
50 KDa
Actin
100 KDa
Flag-FBXW7
75 KDa
Myc-DYRK2
50 KDa
37 KDa
c-Jun
100 KDa
Notch1-IC
50 KDa
c-Myc
Fig. 3F
100 KDa
Flag-FBXW7
75 KDa
DYRK2
100 KDa
Notch1-IC
50 KDa
Actin
Fig. 4A
100 KDa
75 KDa
FBXW7α
100 KDa
75 KDa
DYRK2
Fig. 4C
50 KDa
Actin
100 KDa
Flag-FBXW7
75 KDa
Myc-DYRK2
Fig. 4D
100 KDa
75 KDa
Flag-FBXW7
100 KDa
Flag-FBXW7
75 KDa
Myc-DYRK2
100 KDa
75 KDa
pS/T-FBXW7
50 KDa
Actin
Fig. 5A
100 KDa
Flag-FBXW7
75 KDa
Myc-DYRK2
100 KDa
Flag-FBXW7
75 KDa
Myc-DYRK2
Fig. 5B
100 KDa
Flag-FBXW7
100 KDa
HA-FBXW7
100 KDa
Merge-FBXW7
100 KDa
Flag-FBXW7
75 KDa
Myc-DYRK2
50 KDa
Actin
Fig. 5C
100 KDa
Flag-FBXW7
100 KDa
HA-FBXW7
100 KDa
HA-FBXW7
100 KDa
Flag-FBXW7
50 KDa
Actin
Fig. 6A
75 KDa
DYRK2
100 KDa
Notch1-IC
50 KDa
CyclinE1
250 KDa
mTOR
100 KDa
FBXW7α
75 KDa
50 KDa
c-Myc
50 KDa
37 KDa
c-Jun
50 KDa
Actin
Fig. 6B
75 KDa
DYRK2
100 KDa
FBXW7α
150 KDa
100 KDa
Notch1-IC
50 KDa
CyclinE1
50 KDa
c-Myc
50 KDa
37 KDa
c-Jun
250 KDa
mTOR
50 KDa
Actin
Fig. 6C
75 KDa
DYRK2
50 KDa
Actin
100 KDa
FBXW7α
Fig. 6D
100 KDa
FBXW7α
50 KDa
Actin
75 KDa
DYRK2
Fig. 6E
100 KDa
FBXW7α
75 KDa
DYRK2
100 KDa
FBXW7α
50 KDa
Actin
75 KDa
DYRK2
Fig. 7A
100 KDa
FBXW7α
75 KDa
DYRK2
50 KDa
Actin
Fig. 7D
75 KDa
DYRK2
50 KDa
Actin
100 KDa
FBXW7α
37 KDa
MCL1

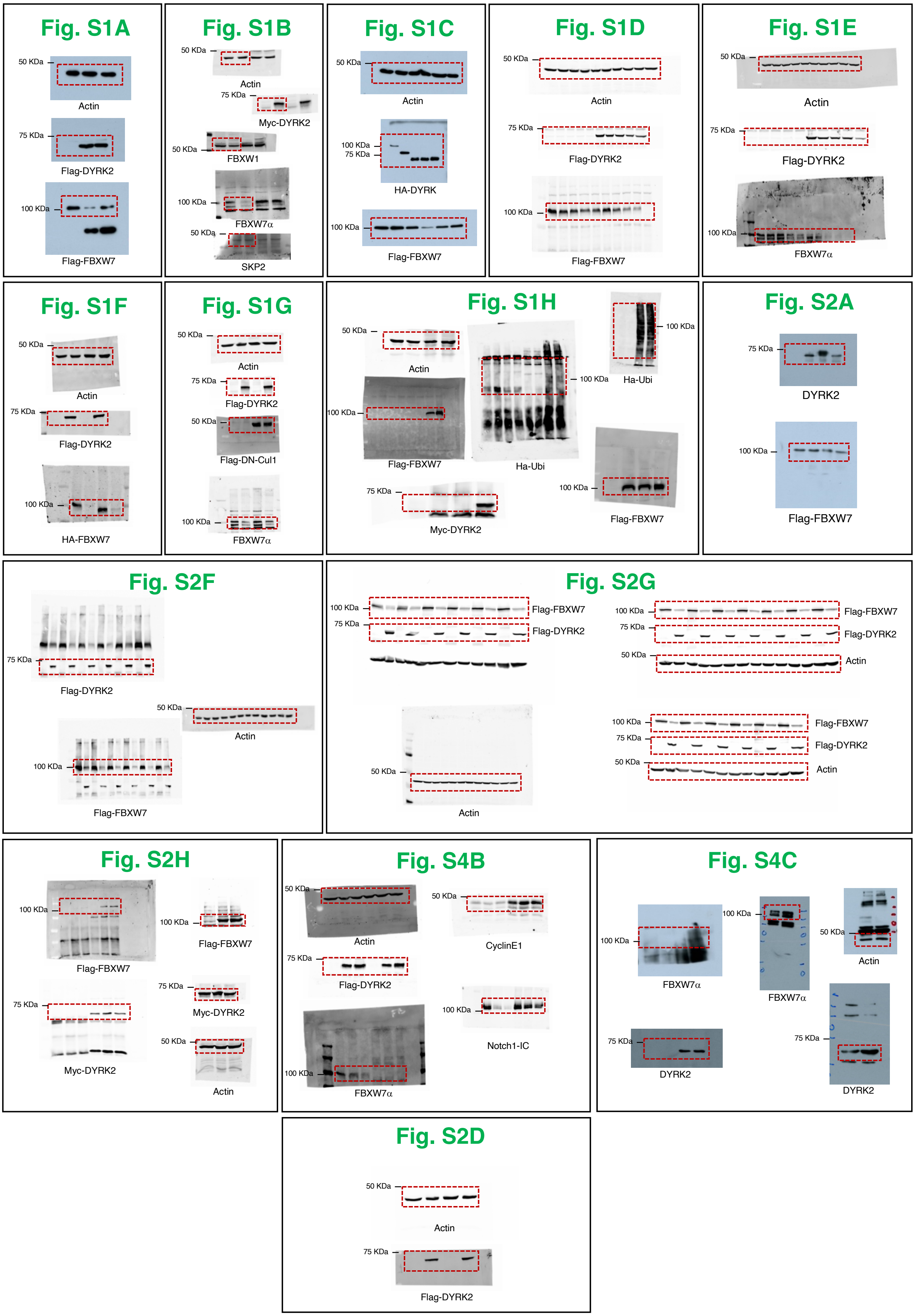
Fig. S1A
50 KDa
Actin
75 KDa
Flag-DYRK2
100 KDa
Flag-FBXW7
Fig. S1B
50 KDa
Actin
75 KDa
Myc-DYRK2
50 KDa
FBXW1
100 KDa
FBXW7α
50 KDa
SKP2
Fig. S1C
50 KDa
Actin
100 KDa
75 KDa
HA-DYRK
100 KDa
Flag-FBXW7
Fig. S1D
50 KDa
Actin
75 KDa
Flag-DYRK2
100 KDa
Flag-FBXW7
Fig. S1E
50 KDa
Actin
75 KDa
Flag-DYRK2
100 KDa
FBXW7α
Fig. S1F
50 KDa
Actin
75 KDa
Flag-DYRK2
100 KDa
HA-FBXW7
Fig. S1G
50 KDa
Actin
75 KDa
Flag-DYRK2
50 KDa
Flag-DN-Cul1
100 KDa
FBXW7α
Fig. S1H
50 KDa
Actin
100 KDa
Flag-FBXW7
75 KDa
Myc-DYRK2
100 KDa
Ha-Ubi
100 KDa
Ha-Ubi
100 KDa
Flag-FBXW7
Fig. S2A
75 KDa
DYRK2
100 KDa
Flag-FBXW7
Fig. S2F
75 KDa
Flag-DYRK2
50 KDa
Actin
100 KDa
Flag-FBXW7
Fig. S2G
100 KDa
Flag-FBXW7
75 KDa
Flag-DYRK2
50 KDa
Actin
100 KDa
Flag-FBXW7
75 KDa
Flag-DYRK2
50 KDa
Actin
100 KDa
Flag-FBXW7
75 KDa
Flag-DYRK2
50 KDa
Actin
Fig. S2H
100 KDa
Flag-FBXW7
100 KDa
Flag-FBXW7
75 KDa
Myc-DYRK2
75 KDa
Myc-DYRK2
50 KDa
Actin
Fig. S4B
50 KDa
Actin
50 KDa
CyclinE1
75 KDa
Flag-DYRK2
100 KDa
Notch1-IC
100 KDa
FBXW7α
Fig. S4C
100 KDa
FBXW7α
100 KDa
FBXW7α
50 KDa
Actin
75 KDa
DYRK2
75 KDa
DYRK2
Fig. S2D
50 KDa
Actin
75 KDa
Flag-DYRK2
